# A survey of practice in management of malignancy-related ascites in Japan

**DOI:** 10.1371/journal.pone.0220869

**Published:** 2019-08-09

**Authors:** Yoshiaki Kanai, Hiroto Ishiki, Isseki Maeda, Satoru Iwase

**Affiliations:** 1 Palliative Care Center, TMG Asaka Medical Center, Saitama, Japan; 2 Division of Palliative Medicine, The University of Tokyo Hospital, Tokyo, Japan; 3 Department of Palliative Medicine, National Cancer Center Hospital, Tokyo, Japan; 4 Gratia Hospice, Gratia Hospital, Osaka, Japan; 5 Department of Palliative Medicine, Saitama Medical University, Saitama, Japan; Cleveland Clinic, UNITED STATES

## Abstract

Although ascites is a distressing complication observed commonly in the course of advanced cancer, there is no effective treatment established for malignancy-related ascites. We conducted a nationwide survey of cancer physicians in Japan who treat malignancy-related ascites in order to determine what kind of therapeutic approach is thought to be significant and what kind of diuretic prescriptions are thought to be standard for malignancy-related ascites. From 2017 to 2018, we sent a one-page memo to oncologists in Japan asking them to participate in a questionnaire-style survey that they could complete online. The significance of each of the nine representative interventions was measured on a 5-stage Likert scale. At the same time, participants were asked about what type and dosage of diuretics they thought to be standard as a treatment for malignancy-related ascites. Ultimately, 187 oncologists responded to our invitation. The interventions that were particularly significant were reducing hydration volume, paracentesis, and symptom management with analgesics. The respondents indicated that the importance of diuretics was significantly lower than that of these three interventions. Furthermore, 86.2% of the respondents in Japan regarded the use of loop diuretics ± aldosterone antagonists as the standard of diuretic therapy for malignancy-related ascites, and the most common regimen was 20 mg of oral furosemide ± 25 mg of spironolactone daily at the start, and 30–40 mg ± 50 mg daily at the time of initial escalation. Although our study revealed that the attitude of oncologists in Japan toward therapeutic options for malignancy-related ascites was nearly consistent with that of previous reports from other countries, it was newly found that they seemed to commonly be concerned with preventing overhydration of terminally ill cancer patients and that analgesics were also thought to be a significant form of intervention.

## Introduction

Ascites is a common complication observed in the course of advanced cancer. One systematic review showed that bloating due to several causes including ascites was observed in 29% (95% CI; 20–40%) of cancer patients whose prognoses exceeded two weeks [1]. In addition, a large volume of ascites causes various types of discomfort such as pain, dyspnea, and early satiety. In one study evaluating the Health-Related Quality of Life (HRQoL) of 103 patients who had ascites due to cirrhosis, the severity of ascites was related to a lower HRQoL [2].

Thus, ascites retention often requires symptomatic relief, but there is no effective treatment established in malignancy-related ascites, and it is often difficult to both reduce ascites and relieve its distress. Although the most readily possible intervention for malignancy-related ascites is diuretic administration, a previous study showed that diuretics had at least a partial benefit in only 26 (38%) of 68 patients [3].

As for why the efficacy of diuretics is inconsistent, it has been pointed out that malignancy-related ascites is a heterogeneous disorder that contains plural mechanisms: peritoneal carcinomatosis and portal hypertension (due to massive hepatoma, cirrhosis or Budd-Chiari syndrome caused by vein occlusion) [4]. Practically, while around 90% of the patients who had malignant ascites were managed by paracentesis, another study showed that only 61% of oncologists use diuretics for their patients with malignant ascites and that only 45% felt them to be effective [5]. Thus, it seems that diuretic treatment is not thought to be as significant as paracentesis for the treatment of cancer-related ascites.

There have been no randomized controlled trials that have evaluated the efficacy and safety of diuretic treatment for cancer-related ascites, nor is there a standard protocol for diuretic usage. At present, oncologists seem to rely on empirical therapy or to follow the guidelines for ascites due to cirrhosis [6]. There have, however, been several reports of oncologists’ preferences in the management of malignancy-related ascites [7–9], yet none are from Asia.

We conducted a nationwide survey of cancer physicians in Japan who treat malignant-related ascites in order to determine what kind of therapeutic approach is thought to be significant and what kind of diuretic prescriptions are thought to be standard for distressing malignancy-related ascites. This is the first report aimed at the trend of supportive care in malignancy-related ascites in Japan.

## Materials and methods

The ethics committee, The University of Tokyo Hospital approved this research. This study was a questionnaire-style survey targeted at medical staff and the data was obtained only from anonymous volunteers. From 2017 to 2018, we sent a memo to physicians involved in cancer treatment in Japan asking them to participate in a questionnaire survey that could be answered online. Oncologists (both physicians and surgeons) practicing anticancer therapy and palliative care physicians practicing symptom management in core oncology centers or at other emergency hospitals were recruited. Clinicians of palliative care units or 24-hour home care clinics also received the invitation. Based on the 344 medical areas defined by the Japanese government, medical facilities were extracted evenly and randomly from each area of Japan.

First, in the questionnaire, the significance of each therapeutic approach (i.e., reducing hydration volume, diuretic administration, albumin administration [including concomitant use with diuretics or paracentesis], paracentesis, concentrated ascites reinfusion therapy [CART] [10, 11], peritoneovenous shunting, antitumor therapy, palliation with corticosteroids, and palliation with analgesics) was measured; we asked the participants whether they thought these approaches were significant or not, and the answers obtained were on a 5-stage Likert scale: 1 = Strongly Disagree, 2 = Disagree, 3 = Neutral, 4 = Agree, and 5 = Strongly Agree.

Second, as a treatment for malignancy-related ascites, participants were asked what kind and dosage of diuretics they thought to be standard. In terms of initial therapy, the physicians were asked to choose one (single agent) or two (in combination) categories from among the following: loop diuretics, aldosterone antagonists, and thiazide diuretics. Next, one or two categories were chosen again for cases involving an increase and/or when the addition of agents was necessary. If the combination of all three categories or another category such as vasopressin receptor antagonists were preferable, the participant was directed to choose "Others" and provide the specifics in the free comment section.

Finally, in the chosen categories, if the physician selected "the standard prescription," we obtained the specific agents and dosages for both initiation and escalation of diuretic therapy from every participant.

Since most of the values obtained via our questionnaires were ordinal variables or non-continuous variables, we used a median as the representative value and tests using a nonparametric technique. To address the problem of multiple tests, we regard all of the statistically significant levels as P < 0.01 throughout this report. The statistical analyses were conducted using Excel Statistics for Windows (Social Survey Research Information, Tokyo, Japan).

## Results

### Characteristics of respondents

In total, 187 oncologists responded to our invitation. The characteristics of the respondents are shown in Table 1. The clinical experience was 24.8 ± 8.5 years (mean ± S.D.), and 29% of the respondents were oncologists practicing anticancer therapy at digestive departments, while 43% were palliative physicians.

**Table 1:** 

Characteristics of respondents (N = 187)	
Experience in clinical medicine (years)	24.8 ± 8.5, Mean ± SD
Gender	Male 174 (93.0%), Female 14 (7.0%)
Types of healthcare facilities	
Governmental cancer centers	13 (7.0%)
Academic hospitals	32 (17.1%)
Other general hospitals	103 (55.1%)
Hospitals not for sufficient acute care	24 (12.8%)
24-hr homecare clinics	15 (8.0%)
Clinical specialty areas	
Digestive system	54 (29.0%)
Respiratory system	10 (5.3%)
Other oncology areas	28 (15.0%)
Supportive care	80 (42.8%)
General practice in hospitals	7 (3.7%)
Homecare	8 (4.3%)
Types of cancer they regard as most problematic	
Gastric	50 (26.7%)
Hepatocellular	30 (16.0%)
Pancreatic	30 (16.0%)
Colorectal	11 (5.9%)
Ovarian	42 (22.5%)
Peritoneal	8 (4.3%)
Others	17 (9.1%)

The most problematic type of cancer in each clinical practice was identified by the respondents: gastric cancer was the most common (26.7%), and this type of cancer along with ovarian, hepatocellular carcinoma, and pancreatic amounted to 81.3%.

### The significance of each intervention

**Fig 1:**
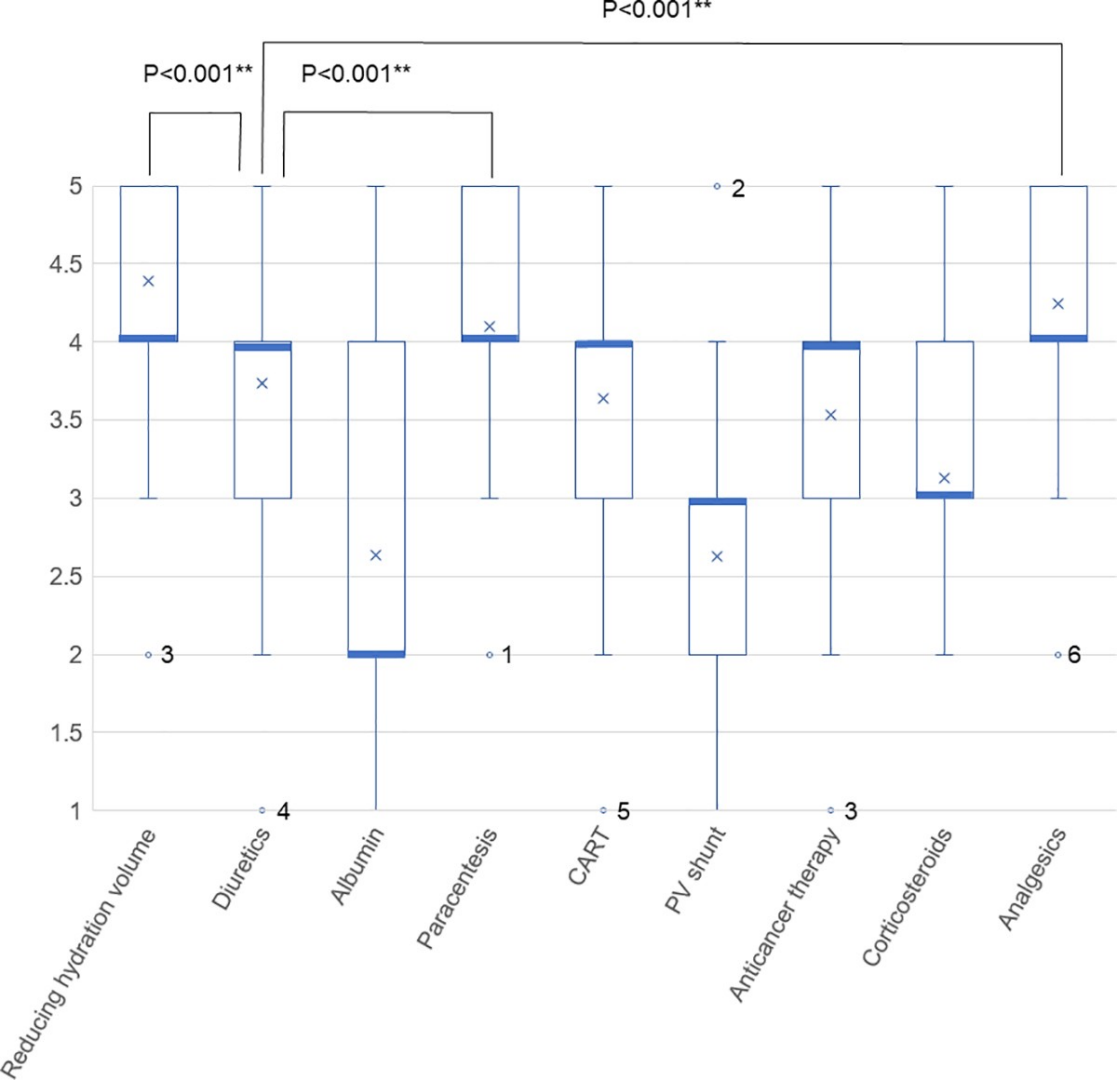
The significance value of each therapeutic approach on a 5-stage Likert scale. Cross marks in the box plots indicate the mean values, the thick lines the median, and the numbers of outliers the respondents who chose that value, respectively. P values were calculated using the Mann-Whitney U-test.

The significance value of each therapeutic approach on a 5-stage Likert scale is shown in (Fig 1). Cross marks in the box plots indicate the mean values, the thick lines the median, and the numbers of outliers the respondents who provided that value. The interventions that were particularly significant were reducing hydration volume, paracentesis, and symptom management with analgesics. The importance of diuretics was significantly lower than that of these three based on a Mann-Whitney’s U-test.

### Identification of the standard notion of diuretic therapy

**Fig 2:**
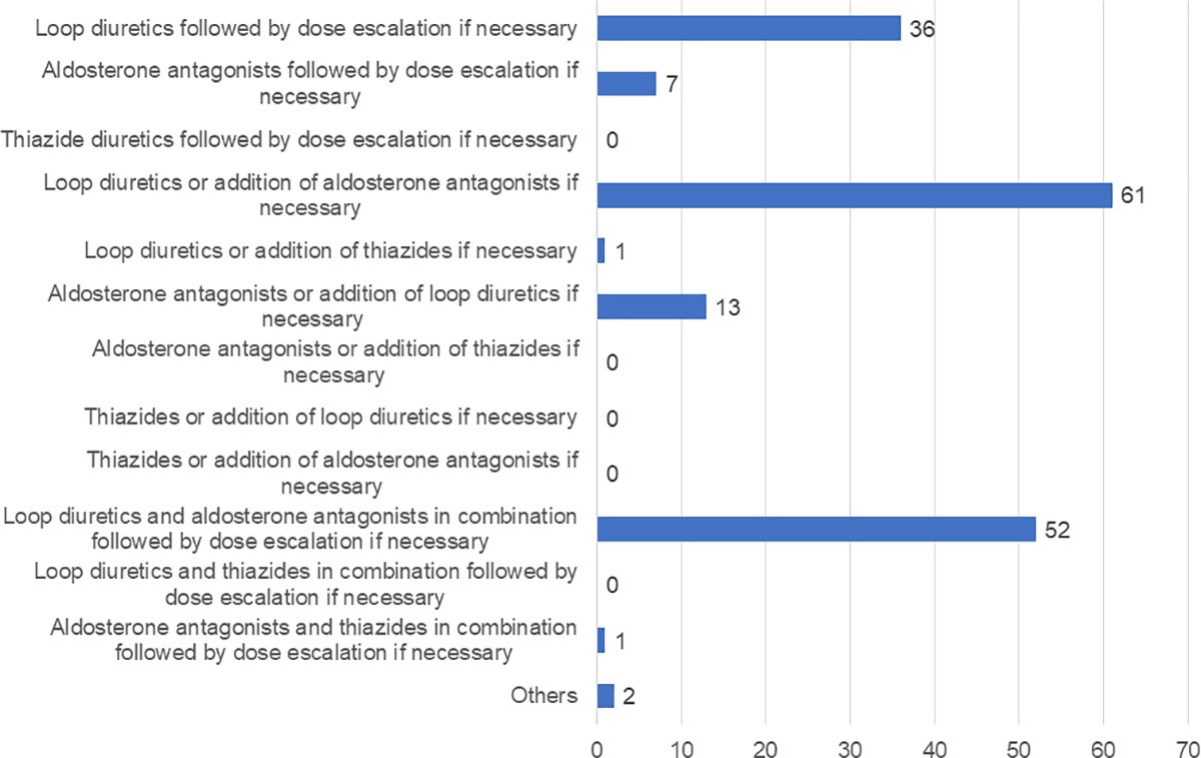
The standard categories of diuretics selected by the respondents. Values indicate the number of physicians who selected each choice. “If necessary” means “when it is needed to add or increase diuretics for the first time”.

The categories of diuretics the respondents believed to be standard when starting them and when it is necessary to add or increase them for the first time are shown in (Fig 2). As the most common opinion, 35.3% of respondents preferred to start with only loop diuretics and add aldosterone antagonists if necessary, 30.1% chose to start with a combination of loop diuretics and aldosterone antagonists and increase both if necessary, and 20.8% chose to start with a loop diuretic single agent and increase if necessary. These three choices accounted for a total of 86.2% of all responses. It was therefore found that most oncologists in Japan regarded the use of loop diuretics ± aldosterone antagonists as the standard of diuretic therapy for malignancy-related ascites.

**Fig 3:**
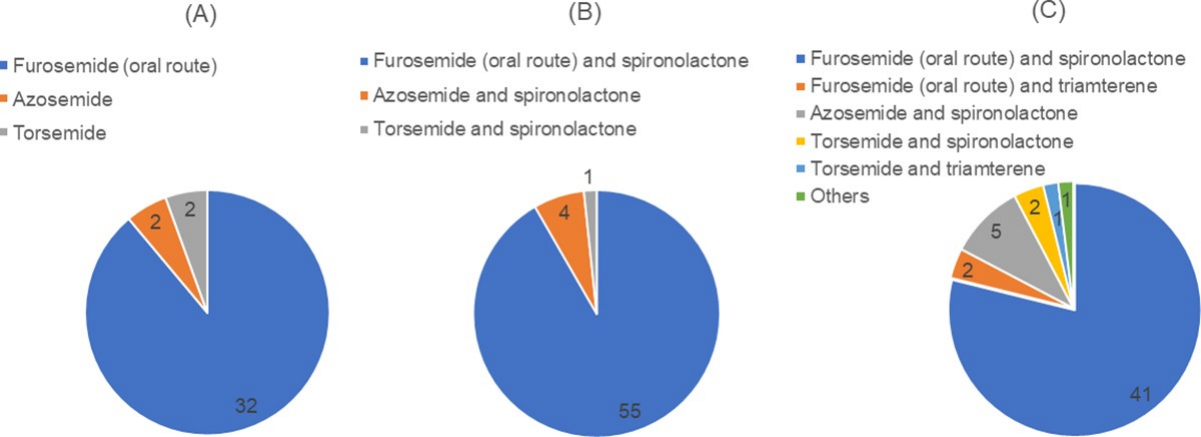
The types of diuretics thought standard by respondents who had chosen loop diuretics ± aldosterone antagonists. (A) The “start with loop diuretics only and increase if necessary” group. (B) The “start with loop diuretics only and add aldosterone antagonists if necessary” group. (C) The “start with a combination of loop diuretics and aldosterone antagonists and increase if necessary” group. For all groups, oral furosemide and spironolactone was the primary choice (P < 0.001**, Chi square test).

Fig 3 shows the types of diuretics believed to be standard by respondents who had chosen loop diuretics ± aldosterone antagonists: 88.9% of the "start with loop diuretics only and increase it necessary" group named oral furosemide, 91.7% of the "start with loop diuretics only and add aldosterone antagonists if necessary" group identified oral furosemide and spironolactone, and 78.9% of the "start with a combination of loop diuretics and aldosterone antagonists and increase if necessary" group named oral furosemide and spironolactone.

**Fig 4:**
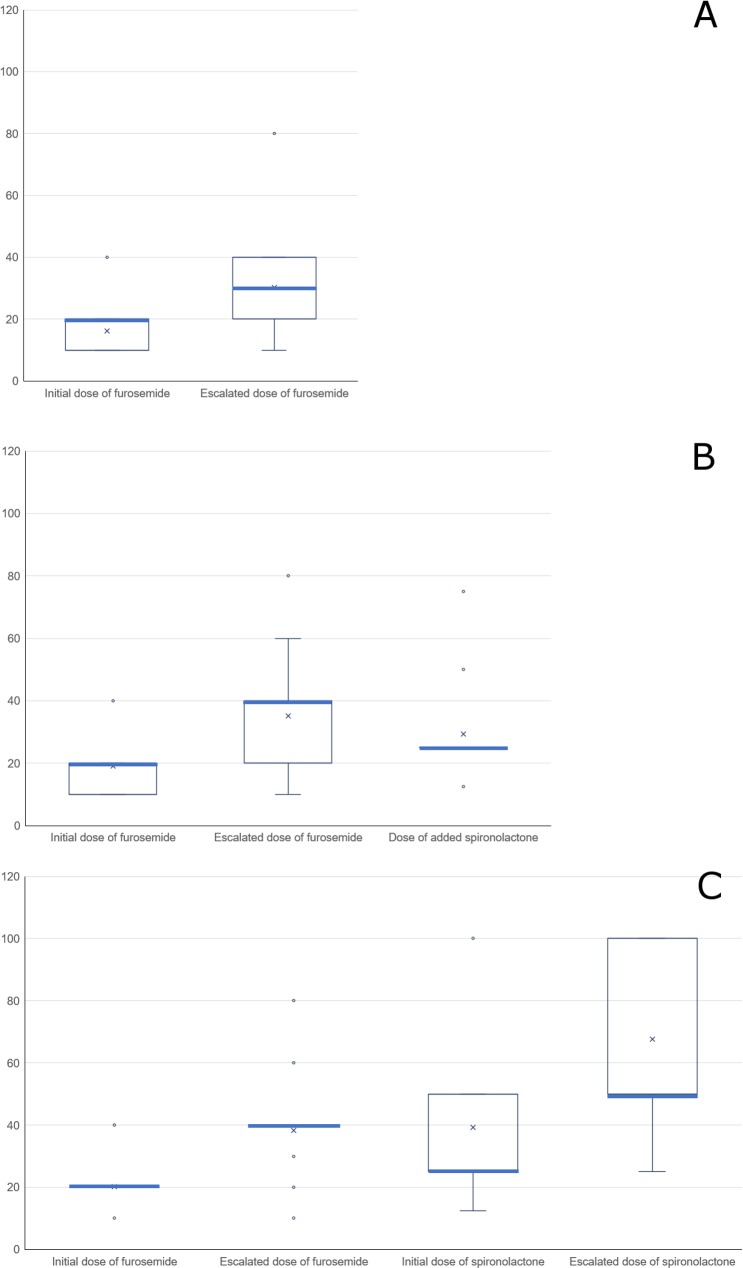
The dosages thought to be standard in each diuretic regimen (mg/day). (A) The “Only furosemide” group (N = 32). (B) The “Only furosemide to add spironolactone” group (N = 55). (C) The “Furosemide + spironolactone” group (N = 41). Cross marks in the box plots indicate the mean values, the thick lines the median, and each dot one outlier, respectively.

Furthermore, in each regimen, the standard dosages used by those who had named them are shown in (Fig 4). In terms of each median value, the starting and escalating doses of oral furosemide (single agent) were 20 mg and 30 mg per day (A); the starting dose of oral furosemide (single agent) was 20 mg, and the escalated furosemide and additional spironolactone were 40 mg and 25 mg per day (B); the dose of furosemide and spironolactone in combination were 20 mg and 25 mg per day at the start and 40 mg and 50 mg per day at the time of escalation (C).

## Discussion/Conclusions

As shown above, it was found that when oncologists in Japan treat cancer-related ascites, reducing hydration volume, paracentesis, and symptom management with analgesics are regarded as the most significant methodologies, and the use of diuretics is thought to be less important. Furthermore, the diuretics thought to be the most standard in the treatment of cancer-related ascites were furosemide ± spironolactone, with the most common daily doses of 20 mg ± 25 mg at the start and of 30–40 mg ± 50 mg at the time of initial escalation.

Our study revealed that the attitude of oncologists in Japan toward therapeutic options for malignancy-related ascites was nearly consistent with that of previous reports from other countries. In a Canadian survey reported in 1998, among the 44 physicians who treat malignant ascites, paracentesis was employed by 43 (98%) and felt to be effective by 39 (89%); diuretics were used by 61% (27/44), although fewer felt diuretics were an effective form of management (20/44, 45%) [7]. A report from the UK published in 2005 showed that most of the respondents prescribed either spironolactone or a combination of spironolactone and furosemide for malignant ascites, sometimes (48.6%) or often (37.2%) [8]. And In a 2015 report targeting physicians in Germany and Austria, while almost all physicians (89%) performed paracentesis at some point in the treatment of malignant ascites, only 55% felt that a concomitant diuretic therapy was a necessary adjunct [9].

Although there has been no sufficient investigation of the efficacy and safety of reducing hydration volume to manage malignancy-related ascites, oncologists in Japan seemed to commonly express concern about preventing overhydration in terminally ill cancer patients. A randomized controlled trial that examined the effects of infusion in terminally ill cancer patients indicated no significant differences in the scores of symptoms, delirium, or fatigue [12]. In contrast, one observational study in Japan suggested that in terminal cancer patients predicted to have less than 3 months of life expectancy, hydration therapy could worsen edema, ascites, and pleural effusions [13].

We also simultaneously investigated what the oncologist thought about using analgesics or corticosteroids in order to improve distress itself [14, 15]. Analgesics were thought to be as significant as paracentesis, but the significance of corticosteroids for the purpose of improving gastrointestinal symptoms and pain associated with ascites retention to restore total wellbeing was inconsistent.

As for the limitations of this study, we could obtain only 187 responses out of totally 1,700 recruitment. Because of the low response rate (11.0%), it is difficult to conclude that these results were representative of oncologists throughout Japan. In addition, although the subspecialties were evenly allocated when extracting participants, the response rate of palliative physicians was approximately double that of oncologists practicing anticancer therapy, which may be due to the difference in interest in symptom management. However, respondents who mainly practice anticancer therapy seemed to agree more strongly with an albumin administration and cytoreductive strategy than did those who mainly practice palliative care (Spearman’s rank correlation coefficients were 0.22 [P<0.01] and 0.31 [P<0.01] respectively), but in terms of reducing hydration, diuretics, and analgesics, no statistically significant differences were observed.

Unfortunately, it is still unclear what therapeutic strategy is best to improve the quality of life or survival of the patients with malignancy-related ascites. Our study revealed only the preference of oncologists in Japan when they treat malignancy-related ascites. Similarly, although our study suggested that oral furosemide and spironolactone were diuretics most commonly used for malignancy-related ascites, it seemed that the majority of respondents chose them not from theoretical grounds for pharmacological action, but they were instead recognized only as "easy-to-use drugs" that oncologists were familiar with in terms of safety and the influence on renal blood flow. It is also possible to agree empirically that the efficacy of diuretics on malignancy-related ascites is limited since its theoretical basis is the result of only one report that investigated the relationship between the cause of ascites and the effect of diuretics of about 16 ascitic cases including 9 that were malignancy-related [16]. It also remains to be clarified how effective novel diuretics such as vasopressin receptor antagonists and their concomitant therapies are in the treatment malignancy-related ascites. Further research is needed for the treatment of cancer-related ascites to become more standardized.

## Supporting information

S1 Questionnaire (PDF)

S2 Questionnaire (PDF)
