# Supplementary material for: A survey of practice in management of malignancy-related ascites in Japan
Source: PLoS One. 2019 Aug 9;14(8):e0220869. doi: 10.1371/journal.pone.0220869 (PMC6688816; doi:10.1371/journal.pone.0220869)
Supplement: S1 Questionnaire — (PDF) [file pone.0220869.s001.pdf]

Survey Questions of the Study; English Translation

Notes:

These were questioned on a website (https://jp.surveymonkey.com) and following is the copied text.

All the participants were required to answer from Q1 to Q78; from Q79 to Q213, we structured the webpages to jump to remaining one or two questions in response to each choice.

Partly questions which had no relation with this report were included.

The mean time of all the participants needed to complete this questionnaire was 12 minutes (measured by the website).

Questionnaire of Practice in Management of Malignancy-Related Ascites

1. Respondent's Characteristics

Q1. How many years have you been a graduate from medical school?

Q2. Please answer your gender.

Male, Female

Q3. Please select all of the following items that are applicable to the medical institution where you mainly treat cancer.

Cancer center

University hospital

Cancer treatment hospital designated by the government

The other general hospital

Hospitals that do not fall under any of the above

24-hour homecare clinic

Other kinds of facilities

Q4. Please answer the number of beds of medical institutions where you mainly treat cancer; truncate less than 100.

Q5. Please choose the closest item to the medical department to which you belong.

Digestive system

Respiratory system

Urology

Gynecology

Breast surgery

Hematology

Oncology
General practice
Supportive care
Homecare
Q6. At medical institutions where you mainly treat cancer, are you primarily in charge of anti-cancer treatment? Or in charge of supportive care? Please choose the closest item to your situation.
Primarily in charge of anti-cancer treatment
Primarily in charge of supportive care
In charge of both equally
Q7. Which of the following is the main setting where you provide symptom relief and healthcare assistance? Please choose the closest item to your situation.
Cross-sectional team providing palliative and supportive care
Acute wards (as a physician in charge)
Palliative care unit
Long-term care facilities
Homecare
Others

Q8. What type of cancer do you regard as most problematic when you treat malignancy-related ascites?
Gastric, Hepatocellular, Pancreatic, Colorectal, Ovarian, Peritoneal, Lung, Prostatic, Breast, Lymphoma, Others

From here, please answer the questions considering about the type of cancer you chose in Q8.

2. Reducing Hydration Volume
Q9. Do you think that reducing hydration volume is significant intervention for malignancy-related ascites?
Strongly agree, Agree, Neutral, Disagree, Strongly disagree
Q10. Do you think that you often reduced hydration volume as the intervention for malignancy-related ascites?
Strongly agree, Agree, Neutral, Disagree, Strongly disagree
Q11. Do you think that reducing hydration volume effectively improves malignancy-related ascites itself?
Strongly agree, Agree, Neutral, Disagree, Strongly disagree

Q12. Do you think that reducing hydration volume effectively improves symptoms due to malignancy-related ascites?

Strongly agree, Agree, Neutral, Disagree, Strongly disagree

Q13. Do you think that reducing hydration volume is easy to choose in the cases with long life expectancy?

Strongly agree, Agree, Neutral, Disagree, Strongly disagree

Q14. Do you think that reducing hydration volume is easy to choose in the cases with short life expectancy?

Strongly agree, Agree, Neutral, Disagree, Strongly disagree

Q15. Do you think that reducing hydration volume is safe intervention in the cases with severe frailty?

Strongly agree, Agree, Neutral, Disagree, Strongly disagree

3. Diuretics

Q16. Do you think that diuretics are significant intervention for malignancy-related ascites?

Strongly agree, Agree, Neutral, Disagree, Strongly disagree

Q17. Do you think that you often prescribed diuretcs as the intervention for malignancy-related ascites?

Strongly agree, Agree, Neutral, Disagree, Strongly disagree

Q18. Do you think that diuretcs effectively improves malignancy-related ascites itself?

Strongly agree, Agree, Neutral, Disagree, Strongly disagree

Q19. Do you think that diuretics effectively improves symptoms due to malignancy-related ascites?

Strongly agree, Agree, Neutral, Disagree, Strongly disagree

Q20. Do you think that diuretics are easy to choose in the cases with long life expectancy?

Strongly agree, Agree, Neutral, Disagree, Strongly disagree

Q21. Do you think that diuretics are easy to choose in the cases with short life expectancy?

Strongly agree, Agree, Neutral, Disagree, Strongly disagree

Q22. Do you think that diuretic therapy is safe intervention in the cases with severe frailty?

Strongly agree, Agree, Neutral, Disagree, Strongly disagree

4. Albumin administration (including combination with other therapies such as diuretics)

Q23. Do you think that albumin administration is significant intervention for malignancy-related ascites?

Strongly agree, Agree, Neutral, Disagree, Strongly disagree

Q24. Do you think that you often administrated albumin as the intervention for malignancy-

related ascites?
Strongly agree, Agree, Neutral, Disagree, Strongly disagree
Q25. Do you think that albumin administration is easy to choose in your facility?
Strongly agree, Agree, Neutral, Disagree, Strongly disagree
Q26. Do you think that transfused albumin effectively improves malignancy-related ascites itself?
Strongly agree, Agree, Neutral, Disagree, Strongly disagree
Q27. Do you think that transfused albumin effectively improves symptoms due to malignancy-related ascites?
Strongly agree, Agree, Neutral, Disagree, Strongly disagree
Q28. Do you think that albumin administration is easy to choose in the cases with long life expectancy?
Strongly agree, Agree, Neutral, Disagree, Strongly disagree
Q29. Do you think that albumin administration is easy to choose in the cases with short life expectancy?
Strongly agree, Agree, Neutral, Disagree, Strongly disagree
Q30. Do you think that albumin administration is safe intervention in the cases with severe frailty?
Strongly agree, Agree, Neutral, Disagree, Strongly disagree

5. Paracentesis (excluding concentrated ascites reinfusion therapy; CART)
Q31. Do you think that paracentesis is significant intervention for malignancy-related ascites?
Strongly agree, Agree, Neutral, Disagree, Strongly disagree
Q32. Do you think that you often performed paracentesis as the intervention for malignancy-related ascites?
Strongly agree, Agree, Neutral, Disagree, Strongly disagree
Q33. Do you think that paracentesis is easy to choose in your facility?
Strongly agree, Agree, Neutral, Disagree, Strongly disagree
Q34. Do you think that paracentesis effectively improves malignancy-related ascites itself?
Strongly agree, Agree, Neutral, Disagree, Strongly disagree
Q35. Do you think that paracentesis effectively improves symptoms due to malignancy-related ascites?
Strongly agree, Agree, Neutral, Disagree, Strongly disagree
Q36. Do you think that paracentesis is easy to choose in the cases with long life expectancy?
Strongly agree, Agree, Neutral, Disagree, Strongly disagree
Q37. Do you think that paracentesis is easy to choose in the cases with short life expectancy?

Strongly agree, Agree, Neutral, Disagree, Strongly disagree

Q38. Do you think that paracentesis is safe intervention in the cases with severe frailty?

Strongly agree, Agree, Neutral, Disagree, Strongly disagree

6. Concentrated Ascites Reinfusion Therapy; CART

Q39. Do you think that CART is significant intervention for malignancy-related ascites?

Strongly agree, Agree, Neutral, Disagree, Strongly disagree

Q40. Do you think that you often performed CART as the intervention for malignancy-related ascites?

Strongly agree, Agree, Neutral, Disagree, Strongly disagree

Q41. Do you think that CART is easy to choose in your facility?

Strongly agree, Agree, Neutral, Disagree, Strongly disagree

Q42. Do you think that CART effectively improves malignancy-related ascites itself?

Strongly agree, Agree, Neutral, Disagree, Strongly disagree

Q43. Do you think that CART effectively improves symptoms due to malignancy-related ascites?

Strongly agree, Agree, Neutral, Disagree, Strongly disagree

Q44. Do you think that CART is easy to choose in the cases with long life expectancy?

Strongly agree, Agree, Neutral, Disagree, Strongly disagree

Q45. Do you think that CART is easy to choose in the cases with short life expectancy?

Strongly agree, Agree, Neutral, Disagree, Strongly disagree

Q46. Do you think that CART is safe intervention in the cases with severe frailty?

Strongly agree, Agree, Neutral, Disagree, Strongly disagree

7. Peritoneovenous Shunt

Q47. Do you think that peritoneovenous shunt is significant intervention for malignancy-related ascites?

Strongly agree, Agree, Neutral, Disagree, Strongly disagree

Q48. Do you think that you often chose peritoneovenous shunts as the intervention for malignancy-related ascites?

Strongly agree, Agree, Neutral, Disagree, Strongly disagree

Q49. Do you think that peritoneovenous shunt is easy to choose in your facility?

Strongly agree, Agree, Neutral, Disagree, Strongly disagree

Q50. Do you think that peritoneovenous shunt effectively improves malignancy-related ascites itself?

Strongly agree, Agree, Neutral, Disagree, Strongly disagree

Q51. Do you think that peritoneovenous shunt effectively improves symptoms due to malignancy-related ascites?

Strongly agree, Agree, Neutral, Disagree, Strongly disagree

Q52. Do you think that peritoneovenous shunt is easy to choose in the cases with long life expectancy?

Strongly agree, Agree, Neutral, Disagree, Strongly disagree

Q53. Do you think that peritoneovenous shunt is easy to choose in the cases with short life expectancy?

Strongly agree, Agree, Neutral, Disagree, Strongly disagree

Q54. Do you think that peritoneovenous shunt is safe intervention in the cases with severe frailty?

Strongly agree, Agree, Neutral, Disagree, Strongly disagree

8. Anticancer Therapy

Q55. Do you think that anticancer therapy is significant intervention for malignancy-related ascites?

Strongly agree, Agree, Neutral, Disagree, Strongly disagree

Q56. Do you think that you often chose anticancer therapy as the intervention for malignancy-related ascites?

Strongly agree, Agree, Neutral, Disagree, Strongly disagree

Q57. Do you think that anticancer therapy is easy to choose in your facility?

Strongly agree, Agree, Neutral, Disagree, Strongly disagree

Q58. Do you think that anticancer therapy effectively improves malignancy-related ascites itself?

Strongly agree, Agree, Neutral, Disagree, Strongly disagree

Q59. Do you think that anticancer therapy effectively improves symptoms due to malignancy-related ascites?

Strongly agree, Agree, Neutral, Disagree, Strongly disagree

Q60. Do you think that anticancer therapy is easy to choose in the cases with long life expectancy?

Strongly agree, Agree, Neutral, Disagree, Strongly disagree

Q61. Do you think that anticancer therapy is easy to choose in the cases with short life expectancy?

Strongly agree, Agree, Neutral, Disagree, Strongly disagree

Q62. Do you think that anticancer therapy is safe intervention in the cases with severe frailty?

Strongly agree, Agree, Neutral, Disagree, Strongly disagree

9. Corticosteroids

Q63. Do you think that corticosteroids are significant intervention for malignancy-related ascites?

Strongly agree, Agree, Neutral, Disagree, Strongly disagree

Q64. Do you think that you often prescribed corticosteroids as the intervention for malignancy-related ascites?

Strongly agree, Agree, Neutral, Disagree, Strongly disagree

Q65. Do you think that corticosteroids are easy to administer in your facility?

Strongly agree, Agree, Neutral, Disagree, Strongly disagree

Q66. Do you think that corticosteroids effectively improve malignancy-related ascites itself?

Strongly agree, Agree, Neutral, Disagree, Strongly disagree

Q67. Do you think that corticosteroids effectively improve symptoms due to malignancy-related ascites?

Strongly agree, Agree, Neutral, Disagree, Strongly disagree

Q68. Do you think that corticosteroids are easy to choose in the cases with long life expectancy?

Strongly agree, Agree, Neutral, Disagree, Strongly disagree

Q69. Do you think that corticosteroids are easy to choose in the cases with short life expectancy?

Strongly agree, Agree, Neutral, Disagree, Strongly disagree

Q70. Do you think that corticosteroids are safe to administer in the cases with severe frailty?

Strongly agree, Agree, Neutral, Disagree, Strongly disagree

10. Analgesics

Q71. Do you think that analgesics are significant intervention for malignancy-related ascites?

Strongly agree, Agree, Neutral, Disagree, Strongly disagree

Q72. Do you think that you often prescribed analgesics as the intervention for malignancy-related ascites?

Strongly agree, Agree, Neutral, Disagree, Strongly disagree

Q73. Do you think that analgesics are easy to administer in your facility?

Strongly agree, Agree, Neutral, Disagree, Strongly disagree

Q74. Do you think that analgesics effectively improve malignancy-related ascites itself?

Strongly agree, Agree, Neutral, Disagree, Strongly disagree

Q75. Do you think that analgesics effectively improve symptoms due to malignancy-related ascites?

Strongly agree, Agree, Neutral, Disagree, Strongly disagree

Q76. Do you think that analgesics are easy to choose in the cases with long life expectancy?

Strongly agree, Agree, Neutral, Disagree, Strongly disagree

Q77. Do you think that analgesics are easy to choose in the cases with short life expectancy?

Strongly agree, Agree, Neutral, Disagree, Strongly disagree

Q78. Do you think that analgesics are safe to administer in the cases with severe frailty?

Strongly agree, Agree, Neutral, Disagree, Strongly disagree

11. The Standard Notion of Diuretic Therapy

From here, please answer about what kind and dosage of diuretics you think to be standard. Please consider that patients have no decrease in blood pressure or renal blood flow.

Q79. Please choose categories of diuretics in terms of initial therapy and for the cases increase and/or addition of agents is necessary.

Loop diuretics followed by dose escalation if necessary (Go to Q80)

Aldosterone antagonists followed by dose escalation if necessary (Go to Q81)

Thiazide diuretics followed by dose escalation if necessary (Go to Q82)

Loop diuretics or addition of aldosterone antagonists if necessary (Go to Q83)

Loop diuretics or addition of thiazides if necessary (Go to Q84)

Aldosterone antagonists or addition of loop diuretics if necessary (Go to Q85)

Aldosterone antagonists or addition of thiazides if necessary (Go to Q86)

Thiazides or addition of loop diuretics if necessary (Go to Q87)

Thiazides or addition of aldosterone antagonists if necessary (Go to Q88)

Loop diuretics and aldosterone antagonists in combination followed by dose escalation if necessary (Go to Q89)

Loop diuretics and thiazides in combination followed by dose escalation if necessary (Go to Q90)

Aldosterone antagonists and thiazides in combination followed by dose escalation if necessary (Go to Q91)

Others (e.g. Start with three kinds of diuretics at the same time; Add vasopressin receptor antagonists.)

Q80. You chose “Loop diuretics followed by dose escalation if necessary”. Please choose the names of diuretics from the followings.

Oral furosemide (Go to Q92)
Azosemide (Go to Q94)
Torsemide (Go to Q96)
Others (Concretely)

Q81. You chose “Aldosterone antagonists followed by dose escalation if necessary”. Please choose the names of diuretics from the followings.
Spironolactone (Go to Q98)
Triamterene (Go to Q100)
Others (Concretely)

Q82. You chose “Thiazide diuretics followed by dose escalation if necessary”. Please choose the names of diuretics from the followings.
Trichlormethiazide (Go to Q102)
Others (Concretely)

Q83. You chose “Loop diuretics or addition of aldosterone antagonists if necessary”. Please choose the names of diuretics from the followings.
Oral furosemide and spironolactone (Go to Q104)
Oral furosemide and triamterene (Go to Q107)
Azosemide and spironolactone (Go to Q110)
Azosemide and triamterene (Go to Q113)
Torsemide and spironolactone (Go to Q116)
Torsemide and triamterene (Go to Q119)
Others (Concretely)

Q84. You chose “Loop diuretics or addition of thiazides if necessary”. Please choose the names of diuretics from the followings.
Oral furosemide and trichlormethiazide (Go to Q122)
Azosemide and trichlormethiazide (Go to Q125)
Torsemide and trichlormethiazide (Go to Q128)
Others (Concretely)

Q85. You chose “Aldosterone antagonists or addition of loop diuretics if necessary”. Please choose the names of diuretics from the followings.
Spironolactone and oral furosemide (Go to Q131)

Spironolactone and azosemide (Go to Q134)
Spironolactone and torsemide (Go to Q137)
Triamterene and oral furosemide (Go to Q140)
Triamterene and azosemide (Go to Q143)
Triamterene and torsemide (Go to Q146)
Others (Concretely)

Q86. You chose "Aldosterone antagonists or addition of thiazides if necessary". Please choose the names of diuretics from the followings.
Spironolactone and trichlormethiazide (Go to Q149)
Triamterene and trichlormethiazide (Go to Q152)
Others (Concretely)

Q87. You chose "Thiazides or addition of loop diuretics if necessary". Please choose the names of diuretics from the followings.
Trichlormethiazide and oral furosemide (Go to Q155)
Trichlormethiazide and azosemide (Go to Q158)
Trichlormethiazide and torsemide (Go to Q161)
Others (Concretely)

Q88. You chose "Thiazides or addition of aldosterone antagonists if necessary". Please choose the names of diuretics from the followings.
Trichlormethiazide and spironolactone (Go to Q164)
Trichlormethiazide and triamterene (Go to Q167)
Others (Concretely)

Q89. You chose "Loop diuretics and aldosterone antagonists in combination followed by dose escalation if necessary". Please choose the names of diuretics from the followings.
Oral furosemide and spironolactone (Go to Q170)
Oral furosemide and triamterene (Go to Q174)
Azosemide and spironolactone (Go to Q178)
Azosemide and triamterene (Go to Q182)
Torsemide and spironolactone (Go to Q186)
Torsemide and triamterene (Go to Q190)
Others (Concretely)

Q90. You chose “Loop diuretics and thiazides in combination followed by dose escalation if necessary”. Please choose the names of diuretics from the followings.
Oral furosemide and trichlormethiazide (Go to Q194)
Azosemide and trichlormethiazide (Go to Q198)
Torsemide and trichlormethiazide (Go to Q202)
Others (Concretely)

Q91. You chose “Aldosterone antagonists and thiazides in combination followed by dose escalation if necessary”. Please choose the names of diuretics from the followings.
Spironolactone and trichlormethiazide (Go to Q206)
Triamterene and trichlormethiazide (Go to Q210)
Others (Concretely)

Q92. Please choose the initial dose of oral furosemide from the followings.
10mg, 20mg, 30mg, 40mg, 50mg, 60mg, 70mg, 80mg, More than 80mg (daily)
Q93. Please choose the escalated dose of oral furosemide from the followings.
10mg, 20mg, 30mg, 40mg, 50mg, 60mg, 70mg, 80mg, More than 80mg (daily)
These are all of the questions. We appreciate you for your time and cooperation.

Q94. Please choose the initial dose of azosemide from the followings.
15mg, 30mg, 45mg, 60mg, 75mg, 90mg, 105mg, 120mg, More than 120mg (daily)
Q95. Please choose the escalated dose of azosemide from the followings.
15mg, 30mg, 45mg, 60mg, 75mg, 90mg, 105mg, 120mg, More than 120mg (daily)
These are all of the questions. We appreciate you for your time and cooperation.

Q96. Please choose the initial dose of torsemide from the followings.
2mg, 4mg, 6mg, 8mg, 10mg, 12mg, 14mg, 16mg, More than 16mg (daily)
Q97. Please choose the escalated dose of torsemide from the followings.
2mg, 4mg, 6mg, 8mg, 10mg, 12mg, 14mg, 16mg, More than 16mg (daily)
These are all of the questions. We appreciate you for your time and cooperation.

Q98. Please choose the initial dose of spironolactone from the followings.
12.5mg, 25mg, 50mg, 75mg, 100mg, More than 100mg (daily)
Q99. Please choose the escalated dose of spironolactone from the followings.
12.5mg, 25mg, 50mg, 75mg, 100mg, More than 100mg (daily)

These are all of the questions. We appreciate you for your time and cooperation.

Q100. Please choose the initial dose of triamterene from the followings.

25mg, 50mg, 100mg, 150mg, 200mg, More than 200mg (daily)

Q101. Please choose the escalated dose of triamterene from the followings

25mg, 50mg, 100mg, 150mg, 200mg, More than 200mg (daily)

These are all of the questions. We appreciate you for your time and cooperation.

Q102. Please choose the initial dose of trichlormethiazide from the followings.

1mg, 2mg, 3mg, 4mg, 5mg, 6m, 7mg, 8mg, More than 8mg (daily)

Q103. Please choose the escalated dose of trichlormethiazide from the followings

1mg, 2mg, 3mg, 4mg, 5mg, 6m, 7mg, 8mg, More than 8mg (daily)

These are all of the questions. We appreciate you for your time and cooperation.

Q104. Please choose the initial dose of oral furosemide from the followings.

10mg, 20mg, 30mg, 40mg, 50mg, 60mg, 70mg, 80mg, More than 80mg (daily)

Q105. Please choose the escalated dose of oral furosemide from the followings.

10mg, 20mg, 30mg, 40mg, 50mg, 60mg, 70mg, 80mg, More than 80mg (daily)

Q106. Please choose the dose of added spironolactone from the followings.

12.5mg, 25mg, 50mg, 75mg, 100mg, More than 100mg (daily)

These are all of the questions. We appreciate you for your time and cooperation.

Q107. Please choose the initial dose of oral furosemide from the followings.

10mg, 20mg, 30mg, 40mg, 50mg, 60mg, 70mg, 80mg, More than 80mg (daily)

Q108. Please choose the escalated dose of oral furosemide from the followings.

10mg, 20mg, 30mg, 40mg, 50mg, 60mg, 70mg, 80mg, More than 80mg (daily)

Q109. Please choose the dose of added triamterene from the followings.

25mg, 50mg, 100mg, 150mg, 200mg, More than 200mg (daily)

These are all of the questions. We appreciate you for your time and cooperation.

Q110. Please choose the initial dose of azosemide from the followings.

15mg, 30mg, 45mg, 60mg, 75mg, 90mg, 105mg, 120mg, More than 120mg (daily)

Q111. Please choose the escalated dose of azosemide from the followings.

15mg, 30mg, 45mg, 60mg, 75mg, 90mg, 105mg, 120mg, More than 120mg (daily)

Q112. Please choose the dose of added spironolactone from the followings.

12.5mg, 25mg, 50mg, 75mg, 100mg, More than 100mg (daily)

These are all of the questions. We appreciate you for your time and cooperation.

Q113. Please choose the initial dose of azosemide from the followings.
15mg, 30mg, 45mg, 60mg, 75mg, 90mg, 105mg, 120mg, More than 120mg (daily)
Q114. Please choose the escalated dose of azosemide from the followings.
15mg, 30mg, 45mg, 60mg, 75mg, 90mg, 105mg, 120mg, More than 120mg (daily)
Q115. Please choose the dose of added triamterene from the followings.
25mg, 50mg, 100mg, 150mg, 200mg, More than 200mg (daily)
These are all of the questions. We appreciate you for your time and cooperation.

Q116. Please choose the initial dose of torsemide from the followings.
2mg, 4mg, 6mg, 8mg, 10mg, 12mg, 14mg, 16mg, More than 16mg (daily)
Q117. Please choose the escalated dose of torsemide from the followings.
2mg, 4mg, 6mg, 8mg, 10mg, 12mg, 14mg, 16mg, More than 16mg (daily)
Q118. Please choose the dose of added spironolactone from the followings.
12.5mg, 25mg, 50mg, 75mg, 100mg, More than 100mg (daily)
These are all of the questions. We appreciate you for your time and cooperation.

Q119. Please choose the initial dose of torsemide from the followings.
2mg, 4mg, 6mg, 8mg, 10mg, 12mg, 14mg, 16mg, More than 16mg (daily)
Q120. Please choose the escalated dose of torsemide from the followings.
2mg, 4mg, 6mg, 8mg, 10mg, 12mg, 14mg, 16mg, More than 16mg (daily)
Q121. Please choose the dose of added triamterene from the followings.
25mg, 50mg, 100mg, 150mg, 200mg, More than 200mg (daily)
These are all of the questions. We appreciate you for your time and cooperation.

Q122. Please choose the initial dose of oral furosemide from the followings.
10mg, 20mg, 30mg, 40mg, 50mg, 60mg, 70mg, 80mg, More than 80mg (daily)
Q123. Please choose the escalated dose of oral furosemide from the followings.
10mg, 20mg, 30mg, 40mg, 50mg, 60mg, 70mg, 80mg, More than 80mg (daily)
Q124. Please choose the dose of added trichlormethiazide from the followings.
1mg, 2mg, 3mg, 4mg, 5mg, 6m, 7mg, 8mg, More than 8mg (daily)
These are all of the questions. We appreciate you for your time and cooperation.

Q125. Please choose the initial dose of azosemide from the followings.
15mg, 30mg, 45mg, 60mg, 75mg, 90mg, 105mg, 120mg, More than 120mg (daily)

Q126. Please choose the escalated dose of azosemide from the followings.
15mg, 30mg, 45mg, 60mg, 75mg, 90mg, 105mg, 120mg, More than 120mg (daily)
Q127. Please choose the dose of added trichlormethiazide from the followings.
1mg, 2mg, 3mg, 4mg, 5mg, 6m, 7mg, 8mg, More than 8mg (daily)
These are all of the questions. We appreciate you for your time and cooperation.

Q128. Please choose the initial dose of torsemide from the followings.
2mg, 4mg, 6mg, 8mg, 10mg, 12mg, 14mg, 16mg, More than 16mg (daily)
Q129. Please choose the escalated dose of torsemide from the followings.
2mg, 4mg, 6mg, 8mg, 10mg, 12mg, 14mg, 16mg, More than 16mg (daily)
Q130. Please choose the dose of added trichlormethiazide from the followings.
1mg, 2mg, 3mg, 4mg, 5mg, 6m, 7mg, 8mg, More than 8mg (daily)
These are all of the questions. We appreciate you for your time and cooperation.

Q131. Please choose the initial dose of spironolactone from the followings.
12.5mg, 25mg, 50mg, 75mg, 100mg, More than 100mg (daily)
Q132. Please choose the escalated dose of spironolactone from the followings.
12.5mg, 25mg, 50mg, 75mg, 100mg, More than 100mg (daily)
Q133. Please choose the dose of added furosemide from the followings.
10mg, 20mg, 30mg, 40mg, 50mg, 60mg, 70mg, 80mg, More than 80mg (daily)
These are all of the questions. We appreciate you for your time and cooperation.

Q134. Please choose the initial dose of spironolactone from the followings.
12.5mg, 25mg, 50mg, 75mg, 100mg, More than 100mg (daily)
Q135. Please choose the escalated dose of spironolactone from the followings.
12.5mg, 25mg, 50mg, 75mg, 100mg, More than 100mg (daily)
Q136. Please choose the dose of added azosemide from the followings.
15mg, 30mg, 45mg, 60mg, 75mg, 90mg, 105mg, 120mg, More than 120mg (daily)
These are all of the questions. We appreciate you for your time and cooperation.

Q137. Please choose the initial dose of spironolactone from the followings.
12.5mg, 25mg, 50mg, 75mg, 100mg, More than 100mg (daily)
Q138. Please choose the escalated dose of spironolactone from the followings.
12.5mg, 25mg, 50mg, 75mg, 100mg, More than 100mg (daily)
Q139. Please choose the dose of added torsemide from the followings.
2mg, 4mg, 6mg, 8mg, 10mg, 12mg, 14mg, 16mg, More than 16mg (daily)

These are all of the questions. We appreciate you for your time and cooperation.

Q140. Please choose the initial dose of triamterene from the followings.

25mg, 50mg, 100mg, 150mg, 200mg, More than 200mg (daily)

Q141. Please choose the escalated dose of triamterene from the followings

25mg, 50mg, 100mg, 150mg, 200mg, More than 200mg (daily)

Q142. Please choose the dose of added furosemide from the followings.

10mg, 20mg, 30mg, 40mg, 50mg, 60mg, 70mg, 80mg, More than 80mg (daily)

These are all of the questions. We appreciate you for your time and cooperation.

Q143. Please choose the initial dose of triamterene from the followings.

25mg, 50mg, 100mg, 150mg, 200mg, More than 200mg (daily)

Q144. Please choose the escalated dose of triamterene from the followings

25mg, 50mg, 100mg, 150mg, 200mg, More than 200mg (daily)

Q145. Please choose the dose of added azosemide from the followings.

15mg, 30mg, 45mg, 60mg, 75mg, 90mg, 105mg, 120mg, More than 120mg (daily)

These are all of the questions. We appreciate you for your time and cooperation.

Q146. Please choose the initial dose of triamterene from the followings.

25mg, 50mg, 100mg, 150mg, 200mg, More than 200mg (daily)

Q147. Please choose the escalated dose of triamterene from the followings

25mg, 50mg, 100mg, 150mg, 200mg, More than 200mg (daily)

Q148. Please choose the dose of added torsemide from the followings.

2mg, 4mg, 6mg, 8mg, 10mg, 12mg, 14mg, 16mg, More than 16mg (daily)

These are all of the questions. We appreciate you for your time and cooperation.

Q149. Please choose the initial dose of spironolactone from the followings.

12.5mg, 25mg, 50mg, 75mg, 100mg, More than 100mg (daily)

Q150. Please choose the escalated dose of spironolactone from the followings.

12.5mg, 25mg, 50mg, 75mg, 100mg, More than 100mg (daily)

Q151. Please choose the dose of added trichlormethiazide from the followings

1mg, 2mg, 3mg, 4mg, 5mg, 6m, 7mg, 8mg, More than 8mg (daily)

These are all of the questions. We appreciate you for your time and cooperation.

Q152. Please choose the initial dose of triamterene from the followings.

25mg, 50mg, 100mg, 150mg, 200mg, More than 200mg (daily)

Q153. Please choose the escalated dose of triamterene from the followings
25mg, 50mg, 100mg, 150mg, 200mg, More than 200mg (daily)
Q154. Please choose the dose of added trichlormethiazide from the followings
1mg, 2mg, 3mg, 4mg, 5mg, 6m, 7mg, 8mg, More than 8mg (daily)
These are all of the questions. We appreciate you for your time and cooperation.

Q155. Please choose the initial dose of trichlormethiazide from the followings.
1mg, 2mg, 3mg, 4mg, 5mg, 6m, 7mg, 8mg, More than 8mg (daily)
Q156. Please choose the escalated dose of trichlormethiazide from the followings
1mg, 2mg, 3mg, 4mg, 5mg, 6m, 7mg, 8mg, More than 8mg (daily)
Q157. Please choose the dose of added furosemide from the followings.
10mg, 20mg, 30mg, 40mg, 50mg, 60mg, 70mg, 80mg, More than 80mg (daily)
These are all of the questions. We appreciate you for your time and cooperation.

Q158. Please choose the initial dose of trichlormethiazide from the followings.
1mg, 2mg, 3mg, 4mg, 5mg, 6m, 7mg, 8mg, More than 8mg (daily)
Q159. Please choose the escalated dose of trichlormethiazide from the followings
1mg, 2mg, 3mg, 4mg, 5mg, 6m, 7mg, 8mg, More than 8mg (daily)
Q160. Please choose the dose of added azosemide from the followings.
15mg, 30mg, 45mg, 60mg, 75mg, 90mg, 105mg, 120mg, More than 120mg (daily)
These are all of the questions. We appreciate you for your time and cooperation.

Q161. Please choose the initial dose of trichlormethiazide from the followings.
1mg, 2mg, 3mg, 4mg, 5mg, 6m, 7mg, 8mg, More than 8mg (daily)
Q162. Please choose the escalated dose of trichlormethiazide from the followings
1mg, 2mg, 3mg, 4mg, 5mg, 6m, 7mg, 8mg, More than 8mg (daily)
Q163. Please choose the dose of added torsemide from the followings.
2mg, 4mg, 6mg, 8mg, 10mg, 12mg, 14mg, 16mg, More than 16mg (daily)
These are all of the questions. We appreciate you for your time and cooperation.

Q164. Please choose the initial dose of trichlormethiazide from the followings.
1mg, 2mg, 3mg, 4mg, 5mg, 6m, 7mg, 8mg, More than 8mg (daily)
Q165. Please choose the escalated dose of trichlormethiazide from the followings
1mg, 2mg, 3mg, 4mg, 5mg, 6m, 7mg, 8mg, More than 8mg (daily)
Q166. Please choose the dose of added spironolactone from the followings.
12.5mg, 25mg, 50mg, 75mg, 100mg, More than 100mg (daily)

These are all of the questions. We appreciate you for your time and cooperation.

Q167. Please choose the initial dose of trichlormethiazide from the followings.

1mg, 2mg, 3mg, 4mg, 5mg, 6m, 7mg, 8mg, More than 8mg (daily)

Q168. Please choose the escalated dose of trichlormethiazide from the followings

1mg, 2mg, 3mg, 4mg, 5mg, 6m, 7mg, 8mg, More than 8mg (daily)

Q169. Please choose the dose of added triamterene from the followings.

25mg, 50mg, 100mg, 150mg, 200mg, More than 200mg (daily)

These are all of the questions. We appreciate you for your time and cooperation.

Q170. Please choose the initial dose of oral furosemide from the followings.

10mg, 20mg, 30mg, 40mg, 50mg, 60mg, 70mg, 80mg, More than 80mg (daily)

Q171. Please choose the initial dose of spironolactone from the followings.

12.5mg, 25mg, 50mg, 75mg, 100mg, More than 100mg (daily)

Q172. Please choose the escalated dose of oral furosemide from the followings.

10mg, 20mg, 30mg, 40mg, 50mg, 60mg, 70mg, 80mg, More than 80mg (daily)

Q173. Please choose the escalated dose of spironolactone from the followings.

12.5mg, 25mg, 50mg, 75mg, 100mg, More than 100mg (daily)

These are all of the questions. We appreciate you for your time and cooperation.

Q174. Please choose the initial dose of oral furosemide from the followings.

10mg, 20mg, 30mg, 40mg, 50mg, 60mg, 70mg, 80mg, More than 80mg (daily)

Q175. Please choose the initial dose of triamterene from the followings.

25mg, 50mg, 100mg, 150mg, 200mg, More than 200mg (daily)

Q176. Please choose the escalated dose of oral furosemide from the followings.

10mg, 20mg, 30mg, 40mg, 50mg, 60mg, 70mg, 80mg, More than 80mg (daily)

Q177. Please choose the escalated dose of triamterene from the followings

25mg, 50mg, 100mg, 150mg, 200mg, More than 200mg (daily)

These are all of the questions. We appreciate you for your time and cooperation.

Q178. Please choose the initial dose of azosemide from the followings.

15mg, 30mg, 45mg, 60mg, 75mg, 90mg, 105mg, 120mg, More than 120mg (daily)

Q179. Please choose the initial dose of spironolactone from the followings.

12.5mg, 25mg, 50mg, 75mg, 100mg, More than 100mg (daily)

Q180. Please choose the escalated dose of azosemide from the followings.

15mg, 30mg, 45mg, 60mg, 75mg, 90mg, 105mg, 120mg, More than 120mg (daily)

Q181. Please choose the escalated dose of spironolactone from the followings.
12.5mg, 25mg, 50mg, 75mg, 100mg, More than 100mg (daily)
These are all of the questions. We appreciate you for your time and cooperation.

Q182. Please choose the initial dose of azosemide from the followings.
15mg, 30mg, 45mg, 60mg, 75mg, 90mg, 105mg, 120mg, More than 120mg (daily)
Q183. Please choose the initial dose of triamterene from the followings.
25mg, 50mg, 100mg, 150mg, 200mg, More than 200mg (daily)
Q184. Please choose the escalated dose of azosemide from the followings.
15mg, 30mg, 45mg, 60mg, 75mg, 90mg, 105mg, 120mg, More than 120mg (daily)
Q185. Please choose the escalated dose of triamterene from the followings
25mg, 50mg, 100mg, 150mg, 200mg, More than 200mg (daily)
These are all of the questions. We appreciate you for your time and cooperation.

Q186. Please choose the initial dose of torsemide from the followings.
2mg, 4mg, 6mg, 8mg, 10mg, 12mg, 14mg, 16mg, More than 16mg (daily)
Q187. Please choose the initial dose of spironolactone from the followings.
12.5mg, 25mg, 50mg, 75mg, 100mg, More than 100mg (daily)
Q188. Please choose the escalated dose of torsemide from the followings.
2mg, 4mg, 6mg, 8mg, 10mg, 12mg, 14mg, 16mg, More than 16mg (daily)
Q189. Please choose the escalated dose of spironolactone from the followings.
12.5mg, 25mg, 50mg, 75mg, 100mg, More than 100mg (daily)
These are all of the questions. We appreciate you for your time and cooperation.

Q190. Please choose the initial dose of torsemide from the followings.
2mg, 4mg, 6mg, 8mg, 10mg, 12mg, 14mg, 16mg, More than 16mg (daily)
Q191. Please choose the initial dose of triamterene from the followings.
25mg, 50mg, 100mg, 150mg, 200mg, More than 200mg (daily)
Q192. Please choose the escalated dose of torsemide from the followings.
2mg, 4mg, 6mg, 8mg, 10mg, 12mg, 14mg, 16mg, More than 16mg (daily)
Q193. Please choose the escalated dose of triamterene from the followings
25mg, 50mg, 100mg, 150mg, 200mg, More than 200mg (daily)
These are all of the questions. We appreciate you for your time and cooperation.

Q194. Please choose the initial dose of oral furosemide from the followings.
10mg, 20mg, 30mg, 40mg, 50mg, 60mg, 70mg, 80mg, More than 80mg (daily)

Q195. Please choose the initial dose of trichlormethiazide from the followings.
1mg, 2mg, 3mg, 4mg, 5mg, 6m, 7mg, 8mg, More than 8mg (daily)
Q196. Please choose the escalated dose of oral furosemide from the followings.
10mg, 20mg, 30mg, 40mg, 50mg, 60mg, 70mg, 80mg, More than 80mg (daily)
Q197. Please choose the escalated dose of trichlormethiazide from the followings
1mg, 2mg, 3mg, 4mg, 5mg, 6m, 7mg, 8mg, More than 8mg (daily)
These are all of the questions. We appreciate you for your time and cooperation.

Q198. Please choose the initial dose of azosemide from the followings.
15mg, 30mg, 45mg, 60mg, 75mg, 90mg, 105mg, 120mg, More than 120mg (daily)
Q199. Please choose the initial dose of trichlormethiazide from the followings.
1mg, 2mg, 3mg, 4mg, 5mg, 6m, 7mg, 8mg, More than 8mg (daily)
Q200. Please choose the escalated dose of azosemide from the followings.
15mg, 30mg, 45mg, 60mg, 75mg, 90mg, 105mg, 120mg, More than 120mg (daily)
Q201. Please choose the escalated dose of trichlormethiazide from the followings
1mg, 2mg, 3mg, 4mg, 5mg, 6m, 7mg, 8mg, More than 8mg (daily)
These are all of the questions. We appreciate you for your time and cooperation.

Q202. Please choose the initial dose of torsemide from the followings.
2mg, 4mg, 6mg, 8mg, 10mg, 12mg, 14mg, 16mg, More than 16mg (daily)
Q203. Please choose the initial dose of trichlormethiazide from the followings.
1mg, 2mg, 3mg, 4mg, 5mg, 6m, 7mg, 8mg, More than 8mg (daily)
Q204. Please choose the escalated dose of torsemide from the followings.
2mg, 4mg, 6mg, 8mg, 10mg, 12mg, 14mg, 16mg, More than 16mg (daily)
Q205. Please choose the escalated dose of trichlormethiazide from the followings
1mg, 2mg, 3mg, 4mg, 5mg, 6m, 7mg, 8mg, More than 8mg (daily)
These are all of the questions. We appreciate you for your time and cooperation.

Q206. Please choose the initial dose of spironolactone from the followings.
12.5mg, 25mg, 50mg, 75mg, 100mg, More than 100mg (daily)
Q207. Please choose the initial dose of trichlormethiazide from the followings.
1mg, 2mg, 3mg, 4mg, 5mg, 6m, 7mg, 8mg, More than 8mg (daily)
Q208. Please choose the escalated dose of spironolactone from the followings.
12.5mg, 25mg, 50mg, 75mg, 100mg, More than 100mg (daily)
Q209. Please choose the escalated dose of trichlormethiazide from the followings
1mg, 2mg, 3mg, 4mg, 5mg, 6m, 7mg, 8mg, More than 8mg (daily)

These are all of the questions. We appreciate you for your time and cooperation.

Q210. Please choose the initial dose of triamterene from the followings.

25mg, 50mg, 100mg, 150mg, 200mg, More than 200mg (daily)

Q211. Please choose the initial dose of trichlormethiazide from the followings.

1mg, 2mg, 3mg, 4mg, 5mg, 6m, 7mg, 8mg, More than 8mg (daily)

Q212. Please choose the escalated dose of triamterene from the followings

25mg, 50mg, 100mg, 150mg, 200mg, More than 200mg (daily)

Q213. Please choose the escalated dose of trichlormethiazide from the followings

1mg, 2mg, 3mg, 4mg, 5mg, 6m, 7mg, 8mg, More than 8mg (daily)

These are all of the questions. We appreciate you for your time and cooperation.
