# Supplementary material for: A survey of practice in management of malignancy-related ascites in Japan
Source: PLoS One. 2019 Aug 9;14(8):e0220869. doi: 10.1371/journal.pone.0220869 (PMC6688816; doi:10.1371/journal.pone.0220869)
Supplement: S2 Questionnaire — (PDF) [file pone.0220869.s002.pdf]

Survey Questions of the Study; Original Language (Japanese)
Notes:
These were questioned on a website (https://jp.surveymonkey.com) and following is the copied text.
All the participants were required to answer from Q1 to Q78; from Q79 to Q213, we structured the webpages to jump to remaining one or two questions in response to each choice.
Partly questions which had no relation with this report were included.
The mean time of all the participants needed to complete this questionnaire was 12 minutes (measured by the website).

がん関連腹水の治療方針に関する質問紙調査

1 回答者の背景

Q1. 先生の卒後年数を半角数字でお教えください。

Q2. 先生の性別をお教えください。

男性，女性

Q3. 以下の項目のうち、先生が主としてがん診療を行う医療機関が該当するものをすべて選んでください。

がんセンター，
大学病院または特定機能病院，
がん診療連携拠点病院または認定がん診療病院，
総合病院または医療センター，
上のいずれにも該当しない病院，
有床診療所または在宅療養支援診療所
上のいずれにも該当しない診療所または施設

Q4. 先生が主としてがん診療を行う医療機関の病床数を、100 の位までの数字でお教えください。

Q5. 先生の診療科・診療内容に最も近いものを 1 つだけ選んでください。

消化器領域，呼吸器領域，泌尿器科，婦人科，乳腺内分泌科，血液内科，腫瘍内科，
総合診療科，一般外科，緩和医療・支持治療科，訪問診療，その他

Q6. 先生が主としてがん診療を行う医療機関において、主に抗がん治療を担当していますか？それとも症状緩和や療養支援を担当していますか？下のうち最も近いものを 1 つお選びください。

主に抗がん治療を担当している

主に症状緩和や療養支援を担当している
どちらも同程度に担当している
Q7. 先生が症状緩和や療養支援を行う医療環境のうち、主な場所は以下のうちどれですか？最も当てはまるものを１つお選びください。
院内緩和ケア（コンサルテーション）チーム,
急性期病棟（主治医として診療）
緩和ケア病棟入院料算定施設
障害者病棟・回復期リハビリテーション病棟・地域包括ケア病棟・その他療養病棟
在宅や施設
その他
Q8. がん診療のなかで、最も腹水が問題になると先生が感じるがん種を 1 つお選びください。
胃がん, 肝細胞癌, 膵癌, 大腸癌, 卵巣癌, 腹膜がん, 肺癌, 前立腺癌, 乳癌, リンパ腫, その他

Q9 より以降の質問には、先生が最もがん関連腹水が問題になると感じるがん種についてお答えください。

2 輸液の減量について
Q9. 輸液の減量は、がん関連腹水治療において、重要だと思いますか？
強くそう思う, まあそう思う, どちらとも言えない, あまりそう思わない, 全くそう思わない
Q10. がん関連腹水に対して、輸液の減量を頻繁に行ってきたと思いますか？
強くそう思う, まあそう思う, どちらとも言えない, あまりそう思わない, 全くそう思わない
Q11. 輸液の減量は、がん関連腹水の減量に有効だと思いますか？
強くそう思う, まあそう思う, どちらとも言えない, あまりそう思わない, 全くそう思わない
Q12. 輸液の減量は、がん関連腹水に伴う苦痛の緩和に有効だと思いますか？
強くそう思う, まあそう思う, どちらとも言えない, あまりそう思わない, 全くそう思わない
Q13. 輸液の減量は、予測される予後が長い場合、選択しやすい治療法だと思いますか？
強くそう思う, まあそう思う, どちらとも言えない, あまりそう思わない, 全くそう思わない
Q14. 輸液の減量は、予測される予後が短い場合、選択しやすい治療法だと思いますか？
強くそう思う, まあそう思う, どちらとも言えない, あまりそう思わない, 全くそう思わな

い
Q15. 輸液の減量は、全身状態の不十分な患者さんにも安全に行える治療だと思いますか？
強くそう思う，まあそう思う，どちらとも言えない，あまりそう思わない，全くそう思わない

3 利尿薬投与について
Q16. 利尿薬投与は、がん関連腹水治療において、重要だと思いますか？
強くそう思う，まあそう思う，どちらとも言えない，あまりそう思わない，全くそう思わない
Q17. がん関連腹水に対して、利尿薬投与を頻繁に行ってきたと思いますか？
強くそう思う，まあそう思う，どちらとも言えない，あまりそう思わない，全くそう思わない
Q18. 利尿薬投与は、がん関連腹水の減量に有効だと思いますか？
強くそう思う，まあそう思う，どちらとも言えない，あまりそう思わない，全くそう思わない
Q19. 利尿薬投与は、がん関連腹水に伴う苦痛の緩和に有効だと思いますか？
強くそう思う，まあそう思う，どちらとも言えない，あまりそう思わない，全くそう思わない
Q20. 利尿薬投与は、予測される予後が長い場合、選択しやすい治療法だと思いますか？
強くそう思う，まあそう思う，どちらとも言えない，あまりそう思わない，全くそう思わない
Q21. 利尿薬投与は、予測される予後が短い場合、選択しやすい治療法だと思いますか？
強くそう思う，まあそう思う，どちらとも言えない，あまりそう思わない，全くそう思わない
Q22. 利尿薬投与は、全身状態の不十分な患者さんにも安全に行える治療だと思いますか？
強くそう思う，まあそう思う，どちらとも言えない，あまりそう思わない，全くそう思わない

4 アルブミン投与*について
*以下、アルブミン投与とは利尿薬などとの併用も含むものとします
Q23. アルブミン投与は、がん関連腹水治療において、重要だと思いますか？
強くそう思う，まあそう思う，どちらとも言えない，あまりそう思わない，全くそう思わない
Q24. がん関連腹水に対して、アルブミン投与を頻繁に行ってきたと思いますか？
強くそう思う，まあそう思う，どちらとも言えない，あまりそう思わない，全くそう思わない

Q25. アルブミン投与は、勤務先の医療機関で行いやすい治療ですか？
強くそう思う，まあそう思う，どちらとも言えない，あまりそう思わない，全くそう思わない

Q26. アルブミン投与は、がん関連腹水の減量に有効だと思いますか？
強くそう思う，まあそう思う，どちらとも言えない，あまりそう思わない，全くそう思わない

Q27. アルブミン投与は、がん関連腹水に伴う苦痛の緩和に有効だと思いますか？
強くそう思う，まあそう思う，どちらとも言えない，あまりそう思わない，全くそう思わない

Q28. アルブミン投与は、予測される予後が長い場合、選択しやすい治療法だと思いますか？
強くそう思う，まあそう思う，どちらとも言えない，あまりそう思わない，全くそう思わない

Q29. アルブミン投与は、予測される予後が短い場合、選択しやすい治療法だと思いますか？
強くそう思う，まあそう思う，どちらとも言えない，あまりそう思わない，全くそう思わない

Q30. アルブミン投与は、全身状態の不十分な患者さんにも安全に行える治療だと思いますか？
強くそう思う，まあそう思う，どちらとも言えない，あまりそう思わない，全くそう思わない

5 腹水穿刺排液（腹水濾過再静注療法は除く）について

Q31. 腹水穿刺排液は、がん関連腹水治療において、重要だと思いますか？
強くそう思う，まあそう思う，どちらとも言えない，あまりそう思わない，全くそう思わない

Q32. がん関連腹水に対して、腹水穿刺排液を頻繁に行ってきたと思いますか？
強くそう思う，まあそう思う，どちらとも言えない，あまりそう思わない，全くそう思わない

Q33. 腹水穿刺排液は、勤務先の医療機関で行いやすい治療ですか？
強くそう思う，まあそう思う，どちらとも言えない，あまりそう思わない，全くそう思わない

Q34. 腹水穿刺排液は、がん関連腹水の減量に有効だと思いますか？
強くそう思う，まあそう思う，どちらとも言えない，あまりそう思わない，全くそう思わない

Q35. 腹水穿刺排液は、がん関連腹水に伴う苦痛の緩和に有効だと思いますか？

強くそう思う，まあそう思う，どちらとも言えない，あまりそう思わない，全くそう思わない
Q36. 腹水穿刺排液は、予測される予後が長い場合、選択しやすい治療法だと思いますか？
強くそう思う，まあそう思う，どちらとも言えない，あまりそう思わない，全くそう思わない
Q37. 腹水穿刺排液は、予測される予後が短い場合、選択しやすい治療法だと思いますか？
強くそう思う，まあそう思う，どちらとも言えない，あまりそう思わない，全くそう思わない
Q38. 腹水穿刺排液は、全身状態の不十分な患者さんにも安全に行える治療だと思いますか？
強くそう思う，まあそう思う，どちらとも言えない，あまりそう思わない，全くそう思わない

6 腹水濾過再静注療法 (CART) について
Q39. 腹水濾過再静注療法 (CART)は、がん関連腹水治療において、重要だと思いますか？
強くそう思う，まあそう思う，どちらとも言えない，あまりそう思わない，全くそう思わない
Q40. がん関連腹水に対して、腹水濾過再静注療法 (CART)を頻繁に行ってきたと思いますか？
強くそう思う，まあそう思う，どちらとも言えない，あまりそう思わない，全くそう思わない
Q41. 腹水濾過再静注療法 (CART)は、勤務先の医療機関で行いやすい治療ですか？
強くそう思う，まあそう思う，どちらとも言えない，あまりそう思わない，全くそう思わない
Q42. 腹水濾過再静注療法 (CART)は、がん関連腹水の減量に有効だと思いますか？
強くそう思う，まあそう思う，どちらとも言えない，あまりそう思わない，全くそう思わない
Q43. 腹水濾過再静注療法 (CART)は、がん関連腹水に伴う苦痛の緩和に有効だと思いますか？
強くそう思う，まあそう思う，どちらとも言えない，あまりそう思わない，全くそう思わない
Q44. 腹水濾過再静注療法 (CART)は、予測される予後が長い場合、選択しやすい治療法だと思いますか？
強くそう思う，まあそう思う，どちらとも言えない，あまりそう思わない，全くそう思わない
Q45. 腹水濾過再静注療法 (CART)は、予測される予後が短い場合、選択しやすい治療法だ

と思いますか？
強くそう思う，まあそう思う，どちらとも言えない，あまりそう思わない，全くそう思わない
Q46. 腹水濾過再静注療法 (CART)は、全身状態の不十分な患者さんにも安全に行える治療だと思いますか？
強くそう思う，まあそう思う，どちらとも言えない，あまりそう思わない，全くそう思わない

7 腹腔ー静脈シャントについて
Q47. 腹腔ー静脈シャントは、がん関連腹水治療において、重要だと思いますか？
強くそう思う，まあそう思う，どちらとも言えない，あまりそう思わない，全くそう思わない
Q48. がん関連腹水に対して、腹腔ー静脈シャントを頻繁に行ってきたと思いますか？
強くそう思う，まあそう思う，どちらとも言えない，あまりそう思わない，全くそう思わない
Q49. 腹腔ー静脈シャントは、勤務先の医療機関で行いやすい治療ですか？
強くそう思う，まあそう思う，どちらとも言えない，あまりそう思わない，全くそう思わない
Q50. 腹腔ー静脈シャントは、がん関連腹水の減量に有効だと思いますか？
強くそう思う，まあそう思う，どちらとも言えない，あまりそう思わない，全くそう思わない
Q51. 腹腔ー静脈シャントは、がん関連腹水に伴う苦痛の緩和に有効だと思いますか？
強くそう思う，まあそう思う，どちらとも言えない，あまりそう思わない，全くそう思わない
Q52. 腹腔ー静脈シャントは、予測される予後が長い場合、選択しやすい治療法だと思いますか？
強くそう思う，まあそう思う，どちらとも言えない，あまりそう思わない，全くそう思わない
Q53. 腹腔ー静脈シャントは、予測される予後が短い場合、選択しやすい治療法だと思いますか？
強くそう思う，まあそう思う，どちらとも言えない，あまりそう思わない，全くそう思わない
Q54. 腹腔ー静脈シャントは、全身状態の不十分な患者さんにも安全に行える治療だと思いますか？
強くそう思う，まあそう思う，どちらとも言えない，あまりそう思わない，全くそう思わない

8 抗腫瘍治療について

Q55. 抗腫瘍治療は、がん関連腹水治療において、重要だと思いますか？

強くそう思う，まあそう思う，どちらとも言えない，あまりそう思わない，全くそう思わない

Q56. がん関連腹水に対して、抗腫瘍治療を頻繁に行ってきたと思いますか？

強くそう思う，まあそう思う，どちらとも言えない，あまりそう思わない，全くそう思わない

Q57. 抗腫瘍治療は、勤務先の医療機関で行いやすい治療ですか？

強くそう思う，まあそう思う，どちらとも言えない，あまりそう思わない，全くそう思わない

Q58. 抗腫瘍治療は、がん関連腹水の減量に有効だと思いますか？

強くそう思う，まあそう思う，どちらとも言えない，あまりそう思わない，全くそう思わない

Q59. 抗腫瘍治療は、がん関連腹水に伴う苦痛の緩和に有効だと思いますか？

強くそう思う，まあそう思う，どちらとも言えない，あまりそう思わない，全くそう思わない

Q60. 抗腫瘍治療は、予測される予後が長い場合、選択しやすい治療法だと思いますか？

強くそう思う，まあそう思う，どちらとも言えない，あまりそう思わない，全くそう思わない

Q61. 抗腫瘍治療は、予測される予後が短い場合、選択しやすい治療法だと思いますか？

強くそう思う，まあそう思う，どちらとも言えない，あまりそう思わない，全くそう思わない

Q62. 抗腫瘍治療は、全身状態の不十分な患者さんにも安全に行える治療だと思いますか？

強くそう思う，まあそう思う，どちらとも言えない，あまりそう思わない，全くそう思わない

9 副腎皮質ステロイド薬投与について

Q63. 副腎皮質ステロイド薬投与は、がん関連腹水治療において、重要だと思いますか？

強くそう思う，まあそう思う，どちらとも言えない，あまりそう思わない，全くそう思わない

Q64. がん関連腹水に対して、副腎皮質ステロイド薬投与を頻繁に行ってきたと思いますか？

強くそう思う，まあそう思う，どちらとも言えない，あまりそう思わない，全くそう思わない

Q65. 副腎皮質ステロイド薬投与は、勤務先の医療機関で行いやすい治療ですか？

強くそう思う，まあそう思う，どちらとも言えない，あまりそう思わない，全くそう思わない

Q66. 副腎皮質ステロイド薬投与は、がん関連腹水の減量に有効だと思いますか？

強くそう思う，まあそう思う，どちらとも言えない，あまりそう思わない，全くそう思わない

Q67. 副腎皮質ステロイド薬投与は、がん関連腹水に伴う苦痛の緩和に有効だと思いますか？

強くそう思う，まあそう思う，どちらとも言えない，あまりそう思わない，全くそう思わない

Q68. 副腎皮質ステロイド薬投与は、予測される予後が長い場合、選択しやすい治療法だと思いますか？

強くそう思う，まあそう思う，どちらとも言えない，あまりそう思わない，全くそう思わない

Q69. 副腎皮質ステロイド薬投与は、予測される予後が短い場合、選択しやすい治療法だと思いますか？

強くそう思う，まあそう思う，どちらとも言えない，あまりそう思わない，全くそう思わない

Q70. 副腎皮質ステロイド薬投与は、全身状態の不十分な患者さんにも安全に行える治療だと思いますか？

強くそう思う，まあそう思う，どちらとも言えない，あまりそう思わない，全くそう思わない

10 鎮痛薬投与について

Q71. 鎮痛薬投与は、がん関連腹水治療において、重要だと思いますか？

強くそう思う，まあそう思う，どちらとも言えない，あまりそう思わない，全くそう思わない

Q72. がん関連腹水に対して、鎮痛薬投与を頻繁に行ってきたと思いますか？

強くそう思う，まあそう思う，どちらとも言えない，あまりそう思わない，全くそう思わない

Q73. 鎮痛薬投与は、勤務先の医療機関で行いやすい治療ですか？

強くそう思う，まあそう思う，どちらとも言えない，あまりそう思わない，全くそう思わない

Q74. 鎮痛薬投与は、がん関連腹水の減量に有効だと思いますか？

強くそう思う，まあそう思う，どちらとも言えない，あまりそう思わない，全くそう思わない

Q75. 鎮痛薬投与は、がん関連腹水に伴う苦痛の緩和に有効だと思いますか？

強くそう思う，まあそう思う，どちらとも言えない，あまりそう思わない，全くそう思わない

Q76. 鎮痛薬投与は、予測される予後が長い場合、選択しやすい治療法だと思いますか？

強くそう思う，まあそう思う，どちらとも言えない，あまりそう思わない，全くそう思わない

Q77. 鎮痛薬投与は、予測される予後が短い場合、選択しやすい治療法だと思いますか？

強くそう思う，まあそう思う，どちらとも言えない，あまりそう思わない，全くそう思わない

Q78. 鎮痛薬投与は、全身状態の不十分な患者さんにも安全に行える治療だと思いますか？

強くそう思う，まあそう思う，どちらとも言えない，あまりそう思わない，全くそう思わない

11 標準的な利尿薬の使用法について

以下の項目では、先生ががん関連腹水に対して最も標準的と考える利尿薬の使用法について教えてください。なお、血圧低下や腎血流量低下はないものと仮定してお答えください。

Q79. がん関連腹水に対する最も標準的な利尿薬の使用法として、開始時の組み合わせと、それで反応が悪い場合の薬剤調整について、以下から選んでください。

ループ利尿薬のみで開始し、必要ならループ利尿薬を増量する（Q80 へ）
抗アルドステロン薬のみで開始し、必要なら抗アルドステロン薬を増量する（Q81 へ）
サイアザイド系利尿薬のみで開始し、必要ならサイアザイド系利尿薬を増量する（Q82 へ）
ループ利尿薬のみで開始し、必要なら抗アルドステロン薬を追加する（Q83 へ）
ループ利尿薬のみで開始し、必要ならサイアザイド系利尿薬を追加する（Q84 へ）
抗アルドステロン薬のみで開始し、必要ならループ利尿薬を追加する（Q85 へ）
抗アルドステロン薬のみで開始し、必要ならサイアザイド系利尿薬を追加する(Q86 へ)
サイアザイド系利尿薬のみで開始し、必要ならループ利尿薬を追加する（Q87 へ）
サイアザイド系利尿薬のみで開始し、必要ならアルドステロン薬を追加する（Q88 へ）
ループ利尿薬とカリウム保持性利尿薬の併用で開始し、必要ならいずれかまたは両方を増量する（Q89 へ）
ループ利尿薬とサイアザイド系利尿薬の併用で開始し、必要ならいずれかまたは両方を増量する（Q90 へ）
抗アルドステロン薬とサイアザイド系利尿薬の併用で開始し、必要ならいずれかまたは両方を増量する（Q91 へ）
その他 ：ループ利尿薬、抗アルドステロン薬、サイアザイド系利尿薬３剤を併用するなど(具体的に)

Q80. ループ利尿薬（フロセミド、アゾセミド、トラセミドなど）のみを使用する場合、何を用いますか？以下から選んでください。
フロセミド内服（Q92 へ）
アゾセミド（Q94 へ）
トラセミド（Q96 へ）
その他（具体的に）

Q81. カリウム保持性利尿薬（＝抗アルドステロン薬:スピロノラクトン、トリアムテレン、カンレノ酸カリウムなど）のみを使用する場合、何を用いますか？以下から選んでください。
スピロノラクトン（Q98 へ）
トリアムテレン（Q100 へ）
その他（具体的に）

Q82. サイアザイド系利尿薬のみを使用する場合、何を用いますか？以下から選んでください。
トリクロルメチアジドを用いる（Q102 へ）
その他（具体的に）

Q83. ループ利尿薬（フロセミド、アゾセミド、トラセミドなど）のみで開始し、反応が悪ければカリウム保持性利尿薬（抗アルドステロン薬；スピロノラクトン、トリアムテレン、カンレノ酸カリウムなど）を追加する場合、何を用いますか？以下から選んでください。
フロセミド内服とスピロノラクトン（Q104 へ）
フロセミド内服とトリアムテレン（Q107 へ）
アゾセミドとスピロノラクトン（Q110 へ）
アゾセミドとトリアムテレン（Q113 へ）
トラセミドとスピロノラクトン（Q116 へ）
トラセミドとトリアムテレン（Q119 へ）
その他（具体的に）

Q84. ループ利尿薬（フロセミド、アゾセミド、トラセミドなど）のみで開始し、反応が悪ければサイアザイド系利尿薬（トリクロルメチアジドなど）を追加する場合、何を用いますか？以下から選んでください。
フロセミド内服とトリクロルメチアジド（Q122 へ）
アゾセミドとトリクロルメチアジド（Q125 へ）
トラセミドとトリクロルメチアジド（Q128 へ）
その他（具体的に）

Q85. カリウム保持性利尿薬（抗アルドステロン薬；スピロノラクトン、トリアムテレン、カンレノ酸カリウムなど）のみで開始し、反応が悪ければループ利尿薬（フロセミド、アゾセミド、トラセミドなど）を追加する場合、何を用いますか？以下から選んでください。
スピロノラクトンとフロセミド（Q131 へ）
スピロノラクトンとアゾセミド（Q134 へ）
スピロノラクトンとトラセミド（Q137 へ）
トリアムテレンとフロセミド（Q140 へ）
トリアムテレンとアゾセミド（Q143 へ）
トリアムテレンとトラセミド（Q146 へ）
その他（具体的に）

Q86. カリウム保持性利尿薬（抗アルドステロン薬；スピロノラクトン、トリアムテレン、カンレノ酸カリウムなど）のみで開始し、反応が悪ければサイアザイド系利尿薬（トリクロルメチアジドなど）を追加する場合、何を用いますか？以下から選んでください。
スピロノラクトンとトリクロルメチアジド（Q149 へ）
トリアムテレンとトリクロルメチアジド（Q152 へ）
その他（具体的に）

Q87. サイアザイド系利尿薬（トリクロルメチアジドなど）のみで開始し、反応が悪ければループ利尿薬（フロセミド、アゾセミド、トラセミドなど）を追加する場合、何を用いますか？以下から選んでください。
トリクロルメチアジドとフロセミド（Q155 へ）
トリクロルメチアジドとアゾセミド（Q158 へ）
トリクロルメチアジドとトラセミド（Q161 へ）
その他（具体的に）

Q88. サイアザイド系利尿薬（トリクロルメチアジドなど）のみで開始し、反応が悪ければカリウム保持性利尿薬（抗アルドステロン薬；スピロノラクトン、トリアムテレン、カンレノ酸カリウムなど）を追加する場合、何を用いますか？以下から選んでください。
トリクロルメチアジドとスピロノラクトン（Q164 へ）
トリクロルメチアジドとトリアムテレン（Q167 へ）
その他（具体的に）

Q89. ループ利尿薬（フロセミド、アゾセミド、トラセミドなど）とカリウム保持性利尿薬（抗アルドステロン薬；スピロノラクトン、トリアムテレン、カンレノ酸カリウムなど）の

併用で開始する場合、何を用いますか？以下から選んでください。
フロセミド内服とスピロノラクトン（Q170 へ）
フロセミド内服とトリアムテレン（Q174 へ）
アゾセミドとスピロノラクトン（Q178 へ）
アゾセミドとトリアムテレン（Q182 へ）
トラセミドとスピロノラクトン（Q186 へ）
トラセミドとトリアムテレン（Q190 へ）
その他（具体的に）

Q90. ループ利尿薬（フロセミド、アゾセミド、トラセミドなど）とサイアザイド系利尿薬（トリクロルメチアジドなど）の併用で開始する場合、何を用いますか？以下から選んでください。
フロセミド内服とトリクロルメチアジド（Q194 へ）
アゾセミドとトリクロルメチアジド（Q198 へ）
トラセミドとトリクロルメチアジド（Q202 へ）
その他（具体的に）

Q91. カリウム保持性利尿薬（抗アルドステロン薬；スピロノラクトン、トリアムテレン、カンレノ酸カリウムなど）とサイアザイド系利尿薬（トリクロルメチアジドなど）の併用で開始する場合、何を用いますか？以下から選んでください。
スピロノラクトンとトリクロルメチアジド（Q206 へ）
トリアムテレンとトリクロルメチアジド（Q210 へ）
その他（具体的に）

Q92. 開始時のフロセミド内服の１日用量を、以下から選んでください。
10mg, 20mg, 30mg, 40mg, 50mg, 60mg, 70mg, 80mg, 80mg より多い
Q93. 最初の変更時のフロセミド内服の１日用量を、以下から選んでください。
10mg, 20mg, 30mg, 40mg, 50mg, 60mg, 70mg, 80mg, 80mg より多い
質問は以上です。ご協力ありがとうございました。

Q94. 開始時のアゾセミドの１日用量を、以下から選んでください。
15mg, 30mg, 45mg, 60mg, 75mg, 90mg, 105mg, 120mg, 120mg より多い
Q95. 最初の変更時のアゾセミドの１日用量を、以下から選んでください。
15mg, 30mg, 45mg, 60mg, 75mg, 90mg, 105mg, 120mg, 120mg より多い
質問は以上です。ご協力ありがとうございました。

Q96. 開始時のトラセミドの１日用量を、以下から選んでください。
2mg, 4mg, 6mg, 8mg, 10mg, 12mg, 14mg, 16mg, 16mg より多い
Q97. 最初の変更時のトラセミドの１日用量を、以下から選んでください。
2mg, 4mg, 6mg, 8mg, 10mg, 12mg, 14mg, 16mg, 16mg より多い
質問は以上です。ご協力ありがとうございました。

Q98. 開始時のスピロノラクトンの１日用量を、以下から選んでください。
12.5mg, 25mg, 50mg, 75mg, 100mg, 100mg より多い
Q99. 最初の変更時のスピロノラクトンの１日用量を、以下から選んでください。
12.5mg, 25mg, 50mg, 75mg, 100mg, 100mg より多い
質問は以上です。ご協力ありがとうございました。

Q100. 開始時のトリアムテレンの１日用量を、以下から選んでください。
25mg, 50mg, 100mg, 150mg, 200mg, 200mg より多い
Q101. 最初の変更時のトリアムテレンの１日用量を、以下から選んでください。
25mg, 50mg, 100mg, 150mg, 200mg, 200mg より多い
質問は以上です。ご協力ありがとうございました。

Q102. 開始時のトリクロルメチアジドの１日用量を、以下から選んでください。
1mg, 2mg, 3mg, 4mg, 5mg, 6m, 7mg, 8mg, 8mg より多い
Q103. 最初の変更時のトリクロルメチアジドの１日用量を、以下から選んでください。
1mg, 2mg, 3mg, 4mg, 5mg, 6m, 7mg, 8mg, 8mg より多い
質問は以上です。ご協力ありがとうございました。

Q104. 開始時のフロセミド内服の１日用量を、以下から選んでください。
10mg, 20mg, 30mg, 40mg, 50mg, 60mg, 70mg, 80mg, 80mg より多い
Q105. 最初の変更時のフロセミド内服の１日用量を、以下から選んでください。
10mg, 20mg, 30mg, 40mg, 50mg, 60mg, 70mg, 80mg, 80mg より多い
Q106. 最初の変更時のスピロノラクトンの１日用量を、以下から選んでください。
12.5mg, 25mg, 50mg, 75mg, 100mg, 100mg より多い
質問は以上です。ご協力ありがとうございました。

Q107. 開始時のフロセミド内服の１日用量を、以下から選んでください。
10mg, 20mg, 30mg, 40mg, 50mg, 60mg, 70mg, 80mg, 80mg より多い
Q108. 最初の変更時のフロセミド内服の１日用量を、以下から選んでください。

10mg, 20mg, 30mg, 40mg, 50mg, 60mg, 70mg, 80mg, 80mg より多い

Q109. 最初の変更時のトリアムテレンの１日用量を、以下から選んでください。

25mg, 50mg, 100mg, 150mg, 200mg, 200mg より多い

質問は以上です。ご協力ありがとうございました。

Q110. 開始時のアゾセミドの１日用量を、以下から選んでください。

15mg, 30mg, 45mg, 60mg, 75mg, 90mg, 105mg, 120mg, 120mg より多い

Q111. 最初の変更時のアゾセミドの１日用量を、以下から選んでください。

15mg, 30mg, 45mg, 60mg, 75mg, 90mg, 105mg, 120mg, 120mg より多い

Q112. 最初の変更時のスピロノラクトンの１日用量を、以下から選んでください。

12.5mg, 25mg, 50mg, 75mg, 100mg, 100mg より多い

質問は以上です。ご協力ありがとうございました。

Q113. 開始時のアゾセミドの１日用量を、以下から選んでください。

15mg, 30mg, 45mg, 60mg, 75mg, 90mg, 105mg, 120mg, 120mg より多い

Q114. 最初の変更時のアゾセミドの１日用量を、以下から選んでください。

15mg, 30mg, 45mg, 60mg, 75mg, 90mg, 105mg, 120mg, 120mg より多い

Q115. 最初の変更時のトリアムテレンの１日用量を、以下から選んでください。

25mg, 50mg, 100mg, 150mg, 200mg, 200mg より多い

質問は以上です。ご協力ありがとうございました。

Q116. 開始時のトラセミドの１日用量を、以下から選んでください。

2mg, 4mg, 6mg, 8mg, 10mg, 12mg, 14mg, 16mg, 16mg より多い

Q117. 最初の変更時のトラセミドの１日用量を、以下から選んでください。

2mg, 4mg, 6mg, 8mg, 10mg, 12mg, 14mg, 16mg, 16mg より多い

Q118. 最初の変更時のスピロノラクトンの１日用量を、以下から選んでください。

12.5mg, 25mg, 50mg, 75mg, 100mg, 100mg より多い

質問は以上です。ご協力ありがとうございました。

Q119. 開始時のトラセミドの１日用量を、以下から選んでください。

2mg, 4mg, 6mg, 8mg, 10mg, 12mg, 14mg, 16mg, 16mg より多い

Q120. 最初の変更時のトラセミドの１日用量を、以下から選んでください。

2mg, 4mg, 6mg, 8mg, 10mg, 12mg, 14mg, 16mg, 16mg より多い

Q121. 最初の変更時のトリアムテレンの１日用量を、以下から選んでください。

25mg, 50mg, 100mg, 150mg, 200mg, 200mg より多い

質問は以上です。ご協力ありがとうございました。

Q122. 開始時のフロセミド内服の１日用量を、以下から選んでください。
10mg, 20mg, 30mg, 40mg, 50mg, 60mg, 70mg, 80mg, 80mg より多い
Q123. 最初の変更時のフロセミド内服の１日用量を、以下から選んでください。
10mg, 20mg, 30mg, 40mg, 50mg, 60mg, 70mg, 80mg, 80mg より多い
Q124. 最初の変更時のトリクロルメチアジドの１日用量を、以下から選んでください。
1mg, 2mg, 3mg, 4mg, 5mg, 6m, 7mg, 8mg, 8mg より多い
質問は以上です。ご協力ありがとうございました。

Q125. 開始時のアゾセミドの１日用量を、以下から選んでください。
15mg, 30mg, 45mg, 60mg, 75mg, 90mg, 105mg, 120mg, 120mg より多い
Q126. 最初の変更時のアゾセミドの１日用量を、以下から選んでください。
15mg, 30mg, 45mg, 60mg, 75mg, 90mg, 105mg, 120mg, 120mg より多い
Q127. 最初の変更時のトリクロルメチアジドの１日用量を、以下から選んでください。
1mg, 2mg, 3mg, 4mg, 5mg, 6m, 7mg, 8mg, 8mg より多い
質問は以上です。ご協力ありがとうございました。

Q128. 開始時のトラセミドの１日用量を、以下から選んでください。
2mg, 4mg, 6mg, 8mg, 10mg, 12mg, 14mg, 16mg, 16mg より多い
Q129. 最初の変更時のトラセミドの１日用量を、以下から選んでください。
2mg, 4mg, 6mg, 8mg, 10mg, 12mg, 14mg, 16mg, 16mg より多い
Q130. 最初の変更時のトリクロルメチアジドの１日用量を、以下から選んでください。
1mg, 2mg, 3mg, 4mg, 5mg, 6m, 7mg, 8mg, 8mg より多い
質問は以上です。ご協力ありがとうございました。

Q131. 開始時のスピロノラクトンの１日用量を、以下から選んでください。
12.5mg, 25mg, 50mg, 75mg, 100mg, 100mg より多い
Q132. 最初の変更時のスピロノラクトンの１日用量を、以下から選んでください。
12.5mg, 25mg, 50mg, 75mg, 100mg, 100mg より多い
Q133. 最初の変更時のフロセミド内服の１日用量を、以下から選んでください。
10mg, 20mg, 30mg, 40mg, 50mg, 60mg, 70mg, 80mg, 80mg より多い
質問は以上です。ご協力ありがとうございました。

Q134. 開始時のスピロノラクトンの１日用量を、以下から選んでください。
12.5mg, 25mg, 50mg, 75mg, 100mg, 100mg より多い
Q135. 最初の変更時のスピロノラクトンの１日用量を、以下から選んでください。

12.5mg, 25mg, 50mg, 75mg, 100mg, 100mg より多い

Q136. 最初の変更時のアゾセミドの１日用量を、以下から選んでください。

15mg, 30mg, 45mg, 60mg, 75mg, 90mg, 105mg, 120mg, 120mg より多い

質問は以上です。ご協力ありがとうございました。

Q137. 開始時のスピロノラクトンの１日用量を、以下から選んでください。

12.5mg, 25mg, 50mg, 75mg, 100mg, 100mg より多い

Q138. 最初の変更時のスピロノラクトンの１日用量を、以下から選んでください。

12.5mg, 25mg, 50mg, 75mg, 100mg, 100mg より多い

Q139. 最初の変更時のトラセミドの１日用量を、以下から選んでください。

2mg, 4mg, 6mg, 8mg, 10mg, 12mg, 14mg, 16mg, 16mg より多い

質問は以上です。ご協力ありがとうございました。

Q140. 開始時のトリアムテレンの１日用量を、以下から選んでください。

25mg, 50mg, 100mg, 150mg, 200mg, 200mg より多い

Q141. 最初の変更時のトリアムテレンの１日用量を、以下から選んでください。

25mg, 50mg, 100mg, 150mg, 200mg, 200mg より多い

Q142. 最初の変更時のフロセミド内服の１日用量を、以下から選んでください。

10mg, 20mg, 30mg, 40mg, 50mg, 60mg, 70mg, 80mg, 80mg より多い

質問は以上です。ご協力ありがとうございました。

Q143. 開始時のトリアムテレンの１日用量を、以下から選んでください。

25mg, 50mg, 100mg, 150mg, 200mg, 200mg より多い

Q144. 最初の変更時のトリアムテレンの１日用量を、以下から選んでください。

25mg, 50mg, 100mg, 150mg, 200mg, 200mg より多い

Q145. 最初の変更時のアゾセミドの１日用量を、以下から選んでください。

15mg, 30mg, 45mg, 60mg, 75mg, 90mg, 105mg, 120mg, 120mg より多い

質問は以上です。ご協力ありがとうございました。

Q146. 開始時のトリアムテレンの１日用量を、以下から選んでください。

25mg, 50mg, 100mg, 150mg, 200mg, 200mg より多い

Q147. 最初の変更時のトリアムテレンの１日用量を、以下から選んでください。

25mg, 50mg, 100mg, 150mg, 200mg, 200mg より多い

Q148. 最初の変更時のトラセミドの１日用量を、以下から選んでください。

2mg, 4mg, 6mg, 8mg, 10mg, 12mg, 14mg, 16mg, 16mg より多い

質問は以上です。ご協力ありがとうございました。

Q149. 開始時のスピロノラクトンの１日用量を、以下から選んでください。
12.5mg, 25mg, 50mg, 75mg, 100mg, 100mg より多い
Q150. 最初の変更時のスピロノラクトンの１日用量を、以下から選んでください。
12.5mg, 25mg, 50mg, 75mg, 100mg, 100mg より多い
Q151. 最初の変更時のトリクロルメチアジドの１日用量を、以下から選んでください。
1mg, 2mg, 3mg, 4mg, 5mg, 6m, 7mg, 8mg, 8mg より多い
質問は以上です。ご協力ありがとうございました。

Q152. 開始時のトリアムテレンの１日用量を、以下から選んでください。
25mg, 50mg, 100mg, 150mg, 200mg, 200mg より多い
Q153. 最初の変更時のトリアムテレンの１日用量を、以下から選んでください。
25mg, 50mg, 100mg, 150mg, 200mg, 200mg より多い
Q154. 最初の変更時のトリクロルメチアジドの１日用量を、以下から選んでください。
1mg, 2mg, 3mg, 4mg, 5mg, 6m, 7mg, 8mg, 8mg より多い
質問は以上です。ご協力ありがとうございました。

Q155. 開始時のトリクロルメチアジドの１日用量を、以下から選んでください。
1mg, 2mg, 3mg, 4mg, 5mg, 6m, 7mg, 8mg, 8mg より多い
Q156. 最初の変更時のトリクロルメチアジドの１日用量を、以下から選んでください。
1mg, 2mg, 3mg, 4mg, 5mg, 6m, 7mg, 8mg, 8mg より多い
Q157. 最初の変更時のフロセミド内服の１日用量を、以下から選んでください。
10mg, 20mg, 30mg, 40mg, 50mg, 60mg, 70mg, 80mg, 80mg より多い
質問は以上です。ご協力ありがとうございました。

Q158. 開始時のトリクロルメチアジドの１日用量を、以下から選んでください。
1mg, 2mg, 3mg, 4mg, 5mg, 6m, 7mg, 8mg, 8mg より多い
Q159. 最初の変更時のトリクロルメチアジドの１日用量を、以下から選んでください。
1mg, 2mg, 3mg, 4mg, 5mg, 6m, 7mg, 8mg, 8mg より多い
Q160. 最初の変更時のアゾセミドの１日用量を、以下から選んでください。
15mg, 30mg, 45mg, 60mg, 75mg, 90mg, 105mg, 120mg, 120mg より多い
質問は以上です。ご協力ありがとうございました。

Q161. 開始時のトリクロルメチアジドの１日用量を、以下から選んでください。
1mg, 2mg, 3mg, 4mg, 5mg, 6m, 7mg, 8mg, 8mg より多い
Q162. 最初の変更時のトリクロルメチアジドの１日用量を、以下から選んでください。

1mg, 2mg, 3mg, 4mg, 5mg, 6m, 7mg, 8mg, 8mg より多い

Q163. 最初の変更時のトラセミドの１日用量を、以下から選んでください。

2mg, 4mg, 6mg, 8mg, 10mg, 12mg, 14mg, 16mg, 16mg より多い

質問は以上です。ご協力ありがとうございました。

Q164. 開始時のトリクロルメチアジドの１日用量を、以下から選んでください。

1mg, 2mg, 3mg, 4mg, 5mg, 6m, 7mg, 8mg, 8mg より多い

Q165. 最初の変更時のトリクロルメチアジドの１日用量を、以下から選んでください。

1mg, 2mg, 3mg, 4mg, 5mg, 6m, 7mg, 8mg, 8mg より多い

Q166. 最初の変更時のスピロノラクトンの１日用量を、以下から選んでください。

12.5mg, 25mg, 50mg, 75mg, 100mg, 100mg より多い

質問は以上です。ご協力ありがとうございました。

Q167. 開始時のトリクロルメチアジドの１日用量を、以下から選んでください。

1mg, 2mg, 3mg, 4mg, 5mg, 6m, 7mg, 8mg, 8mg より多い

Q168. 最初の変更時のトリクロルメチアジドの１日用量を、以下から選んでください。

1mg, 2mg, 3mg, 4mg, 5mg, 6m, 7mg, 8mg, 8mg より多い

Q169. 最初の変更時のトリアムテレンの１日用量を、以下から選んでください。

25mg, 50mg, 100mg, 150mg, 200mg, 200mg より多い

質問は以上です。ご協力ありがとうございました。

Q170. 開始時のフロセミド内服の１日用量を、以下から選んでください。

10mg, 20mg, 30mg, 40mg, 50mg, 60mg, 70mg, 80mg, 80mg より多い

Q171. 開始時のスピロノラクトンの１日用量を、以下から選んでください。

12.5mg, 25mg, 50mg, 75mg, 100mg, 100mg より多い

Q172. 最初の変更時のフロセミド内服の１日用量を、以下から選んでください。

10mg, 20mg, 30mg, 40mg, 50mg, 60mg, 70mg, 80mg, 80mg より多い

Q173. 最初の変更時のスピロノラクトンの１日用量を、以下から選んでください。

12.5mg, 25mg, 50mg, 75mg, 100mg, 100mg より多い

質問は以上です。ご協力ありがとうございました。

Q174. 開始時のフロセミド内服の１日用量を、以下から選んでください。

10mg, 20mg, 30mg, 40mg, 50mg, 60mg, 70mg, 80mg, 80mg より多い

Q175. 開始時のトリアムテレンの１日用量を、以下から選んでください。

25mg, 50mg, 100mg, 150mg, 200mg, 200mg より多い

Q176. 最初の変更時のフロセミド内服の１日用量を、以下から選んでください。

10mg, 20mg, 30mg, 40mg, 50mg, 60mg, 70mg, 80mg, 80mg より多い

Q177. 最初の変更時のトリアムテレンの１日用量を、以下から選んでください。

25mg, 50mg, 100mg, 150mg, 200mg, 200mg より多い

質問は以上です。ご協力ありがとうございました。

Q178. 開始時のアゾセミドの１日用量を、以下から選んでください。

15mg, 30mg, 45mg, 60mg, 75mg, 90mg, 105mg, 120mg, 120mg より多い

Q179. 開始時のスピロノラクトンの１日用量を、以下から選んでください。

12.5mg, 25mg, 50mg, 75mg, 100mg, 100mg より多い

Q180. 最初の変更時のアゾセミドの１日用量を、以下から選んでください。

15mg, 30mg, 45mg, 60mg, 75mg, 90mg, 105mg, 120mg, 120mg より多い

Q181. 最初の変更時のスピロノラクトンの１日用量を、以下から選んでください。

12.5mg, 25mg, 50mg, 75mg, 100mg, 100mg より多い

質問は以上です。ご協力ありがとうございました。

Q182. 開始時のアゾセミドの１日用量を、以下から選んでください。

15mg, 30mg, 45mg, 60mg, 75mg, 90mg, 105mg, 120mg, 120mg より多い

Q183. 開始時のトリアムテレンの１日用量を、以下から選んでください。

25mg, 50mg, 100mg, 150mg, 200mg, 200mg より多い

Q184. 最初の変更時のアゾセミドの１日用量を、以下から選んでください。

15mg, 30mg, 45mg, 60mg, 75mg, 90mg, 105mg, 120mg, 120mg より多い

Q185. 最初の変更時のトリアムテレンの１日用量を、以下から選んでください。

25mg, 50mg, 100mg, 150mg, 200mg, 200mg より多い

質問は以上です。ご協力ありがとうございました。

Q186. 開始時のトラセミドの１日用量を、以下から選んでください。

2mg, 4mg, 6mg, 8mg, 10mg, 12mg, 14mg, 16mg, 16mg より多い

Q187. 開始時のスピロノラクトンの１日用量を、以下から選んでください。

12.5mg, 25mg, 50mg, 75mg, 100mg, 100mg より多い

Q188. 最初の変更時のトラセミドの１日用量を、以下から選んでください。

2mg, 4mg, 6mg, 8mg, 10mg, 12mg, 14mg, 16mg, 16mg より多い

Q189. 最初の変更時のスピロノラクトンの１日用量を、以下から選んでください。

12.5mg, 25mg, 50mg, 75mg, 100mg, 100mg より多い

質問は以上です。ご協力ありがとうございました。

Q190. 開始時のトラセミドの１日用量を、以下から選んでください。

2mg, 4mg, 6mg, 8mg, 10mg, 12mg, 14mg, 16mg, 16mg より多い

Q191. 開始時のトリアムテレンの１日用量を、以下から選んでください。

25mg, 50mg, 100mg, 150mg, 200mg, 200mg より多い

Q192. 最初の変更時のトラセミドの１日用量を、以下から選んでください。

2mg, 4mg, 6mg, 8mg, 10mg, 12mg, 14mg, 16mg, 16mg より多い

Q193. 最初の変更時のトリアムテレンの１日用量を、以下から選んでください。

25mg, 50mg, 100mg, 150mg, 200mg, 200mg より多い

質問は以上です。ご協力ありがとうございました。

Q194. 開始時のフロセミド内服の１日用量を、以下から選んでください。

10mg, 20mg, 30mg, 40mg, 50mg, 60mg, 70mg, 80mg, 80mg より多い

Q195. 開始時のトリクロルメチアジドの１日用量を、以下から選んでください。

1mg, 2mg, 3mg, 4mg, 5mg, 6m, 7mg, 8mg, 8mg より多い

Q196. 最初の変更時のフロセミド内服の１日用量を、以下から選んでください。

10mg, 20mg, 30mg, 40mg, 50mg, 60mg, 70mg, 80mg, 80mg より多い

Q197. 最初の変更時のトリクロルメチアジドの１日用量を、以下から選んでください。

1mg, 2mg, 3mg, 4mg, 5mg, 6m, 7mg, 8mg, 8mg より多い

質問は以上です。ご協力ありがとうございました。

Q198. 開始時のアゾセミドの１日用量を、以下から選んでください。

15mg, 30mg, 45mg, 60mg, 75mg, 90mg, 105mg, 120mg, 120mg より多い

Q199. 開始時のトリクロルメチアジドの１日用量を、以下から選んでください。

1mg, 2mg, 3mg, 4mg, 5mg, 6m, 7mg, 8mg, 8mg より多い

Q200. 最初の変更時のアゾセミドの１日用量を、以下から選んでください。

15mg, 30mg, 45mg, 60mg, 75mg, 90mg, 105mg, 120mg, 120mg より多い

Q201. 最初の変更時のトリクロルメチアジドの１日用量を、以下から選んでください。

1mg, 2mg, 3mg, 4mg, 5mg, 6m, 7mg, 8mg, 8mg より多い

質問は以上です。ご協力ありがとうございました。

Q202. 開始時のトラセミドの１日用量を、以下から選んでください。

2mg, 4mg, 6mg, 8mg, 10mg, 12mg, 14mg, 16mg, 16mg より多い

Q203. 開始時のトリクロルメチアジドの１日用量を、以下から選んでください。

1mg, 2mg, 3mg, 4mg, 5mg, 6m, 7mg, 8mg, 8mg より多い

Q204. 最初の変更時のトラセミドの１日用量を、以下から選んでください。

2mg, 4mg, 6mg, 8mg, 10mg, 12mg, 14mg, 16mg, 16mg より多い

Q205. 最初の変更時のトリクロルメチアジドの１日用量を、以下から選んでください。

1mg, 2mg, 3mg, 4mg, 5mg, 6m, 7mg, 8mg, 8mg より多い
質問は以上です。ご協力ありがとうございました。

Q206. 開始時のスピロノラクトンの１日用量を、以下から選んでください。
12.5mg, 25mg, 50mg, 75mg, 100mg, 100mg より多い
Q207. 開始時のトリクロルメチアジドの１日用量を、以下から選んでください。
1mg, 2mg, 3mg, 4mg, 5mg, 6m, 7mg, 8mg, 8mg より多い
Q208. 最初の変更時のスピロノラクトンの１日用量を、以下から選んでください。
12.5mg, 25mg, 50mg, 75mg, 100mg, 100mg より多い
Q209. 最初の変更時のトリクロルメチアジドの１日用量を、以下から選んでください。
1mg, 2mg, 3mg, 4mg, 5mg, 6m, 7mg, 8mg, 8mg より多い
質問は以上です。ご協力ありがとうございました。

Q210. 開始時のトリアムテレンの１日用量を、以下から選んでください。
25mg, 50mg, 100mg, 150mg, 200mg, 200mg より多い
Q211. 開始時のトリクロルメチアジドの１日用量を、以下から選んでください。
1mg, 2mg, 3mg, 4mg, 5mg, 6m, 7mg, 8mg, 8mg より多い
Q212. 最初の変更時のトリアムテレンの１日用量を、以下から選んでください。
25mg, 50mg, 100mg, 150mg, 200mg, 200mg より多い
Q213. 最初の変更時のトリクロルメチアジドの１日用量を、以下から選んでください。
1mg, 2mg, 3mg, 4mg, 5mg, 6m, 7mg, 8mg, 8mg より多い
質問は以上です。ご協力ありがとうございました。
